# Age-related lung changes linked to altered lysosomal protease profile, histology, and ultrastructure

**DOI:** 10.1371/journal.pone.0311760

**Published:** 2024-12-20

**Authors:** Mohammed Aufy, Mahmoud Abd-Elkareem, Medina Mustafic, Mostafa A. Abdel-Maksoud, Ali Hakamy, Veronika Baresova, Akram A. Alfuraydi, Mahmoud Ashry, Jana Lubec, Ayman S. Amer, Christian R. Studenik, Ahmed M. Hussein, Mohamed H. Kotob

**Affiliations:** 1 Division of Pharmacology and Toxicology, Department of Pharmaceutical Sciences, University of Vienna, Vienna, Austria; 2 Department of Cell and Tissue, Faculty of Veterinary Medicine, Assiut University, Assiut, Egypt; 3 Department of Botany and Microbiology, College of Science, King Saud University, Riyadh, Saudi Arabia; 4 Respiratory Therapy Department, Faculty of Applied Medical Sciences, Jazan University, Jazan, Saudi Arabia; 5 Faculty of Pharmacy in Hradec Kralove, Charles University, Prague, Czech Republic; 6 Department of Zoology, Faculty of Science, Al-Azhar University, Assiut, Egypt; 7 Programme for Proteomics, Paracelsus Medical University, Salzburg, Austria; 8 Department of Biomedical Sciences, College of Medicine, King Faisal University, Al Ahsa, Saudi Arabia; 9 Department of Pathology, Faculty of Veterinary Medicine, Assiut University, Assiut, Egypt; Università degli Studi della Campania, ITALY

## Abstract

**Introduction:**

The aging process is intricately linked to alterations in cellular and tissue structures, with the respiratory system being particularly susceptible to age-related changes. Therefore, this study aimed to profile the activity of proteases using activity-based probes in lung tissues of old and young rats, focusing on the expression levels of different, in particular cathepsins G and X and matrix Metalloproteinases (MMPs). Additionally, the impact on extracellular matrix (ECM) components, particularly fibronectin, in relation to age-related histological and ultrastructural changes in lung tissues was investigated.

**Materials and methods:**

Lung tissues from old and young rats were subjected to activity-based probe profiling to assess the activity of different proteases. Expression levels of cathepsins G and X were quantified, and zymography was performed to evaluate matrix metalloproteinases activity. Furthermore, ECM components, specifically fibronectin, were examined for signs of degradation in the old lung tissues compared to the young ones. Moreover, histological, immunohistochemical and ultrastructural assessments of old and young lung tissue were also conducted.

**Results:**

Our results showed that the expression levels of cathepsins G and X were notably higher in old rat lung tissues in contrast to those in young rat lung tissues. Zymography analysis revealed elevated MMP activity in the old lung tissues compared to the young ones. Particularly, significant degradation of fibronectin, an essential ECM component, was observed in the old lung tissues. Numerous histological and ultrastructural alterations were observed in old lung tissues compared to young lung tissues.

**Discussion and conclusion:**

The findings indicate an age-related upregulation of cathepsins G and X along with heightened MMP activity in old rat lung tissues, potentially contributing to the degradation of fibronectin within the ECM. These alterations highlight potential mechanisms underlying age-associated changes in lung tissue integrity and provide insights into protease-mediated ECM remodeling in the context of aging lungs.

## 1. Introduction

The aging process is an inevitable and intricate biological phenomenon that affects nearly every aspect of human physiology.

Lung aging is associated with molecular, structural and functional alterations [1]. These modifications cause decreased respiratory function, pulmonary remodeling, restricted regeneration, and an increased vulnerability to pulmonary diseases [2, 3]. Age-related alterations of the lung mainly include an increase in collagen production, a decrease in elastin production by fibroblasts, a reduction in the number of bronchoepithelial cells and mucus production, compromised mucociliary clearance and increased oxidative stress, what in turn results in an elevated pulmonary stiffness, reduced pulmonary elasticity and reduced ability to effectively combat infectious diseases such as COVID19 [3, 4].

Pulmonary senescence is associated with several diseases, including Chronic Obstructive Pulmonary Disease (COPD), idiopathic pulmonary fibrosis (IPF), lung cancer, and pulmonary hypertension. Aging-related changes in lung structure and function contribute to increased susceptibility to infections, like pneumonia, and conditions such as asthma and interstitial lung diseases [2, 4].

Cathepsins (caths), a family of lysosomal proteases, are known for their diverse roles in cellular homeostasis, immune regulation and response, energy metabolism and extracellular matrix remodeling [5–7]. However, their specific involvement in the aging of the lung remains a topic of burgeoning interest and investigation. Cathepsins have gained a reputation as key players in matrix degradation; however, their importance extends far beyond this fundamental role, and exerts a significant impact on the pathophysiology of pulmonary diseases [8–10]. They are involved in many vital biological processes, including angiogenesis, regulating the availability of growth factors, processing cytokines, shedding and activating receptors, as well as modulating cellular functions like migration, proliferation, invasion, and survival [11]. Among almost 50 lysosomal hydrolases identified, the aspartic, serine, and cysteine proteases are particularly noteworthy [12–14]. Notably, research has revealed the involvement of the serine protease cath G and the cysteine proteases cath X and calpain in aging processes [15–21].

Our investigation aims to delve deeper into the molecular mechanisms underlying the complex interplay between cathepsins and the aging lung. While the intricate details of this relationship remain to be unraveled, emerging evidence suggests that alterations in cathepsins activity may be instrumental in shaping the physiological changes observed in the aging respiratory system [22]. The activation of calpain might lead to significant cellular alterations associated with aging and/or atrial fibrillation (AF). Studies have shown that calpains play a role in the degradation of proteins related to cytoskeletal structure, contractility, and L-type Ca2+ channels, contributing to atrial remodeling in AF [23, 24].

Cath X has been recognized for its significant involvement in inflammatory processes. Furthermore, an upregulation of this enzyme in the aging mouse brain, along with mouse models of neurodegenerative diseases like amyotrophic lateral sclerosis (ALS) and Alzheimer’s disease (AD) has also been reported [17].

Cath G stands out as one of the principal serine proteases secreted from the azurophilic granules of neutrophils. Beyond its well-known antibacterial properties, cath G serves a multifaceted role in innate immunity, chemoattraction, and the degradation of the extracellular matrix [25]. Intriguingly, studies have shown that cath G can exhibit both protective and detrimental effects on lung health, depending on the context.

In some instances, cath G has been found to be a defender of lung tissue integrity. For example, it has been shown to play a protective role against lung damage induced by infections such as *Streptococcus pneumoniae* [26]. On the other hand, there is evidence to suggest that reducing cath G levels through genetic knockdown can shield lung tissue from the destructive effects of long-term exposure to cigarette smoke. Furthermore, an upsurge in cath G expression has been associated with adverse outcomes in chronic obstructive pulmonary disease (COPD) patients. This increased expression has been linked to alveolar wall damage and the abnormal secretion of mucus from the airway serous cells [27, 28] highlighting its potential involvement in the pathogenesis of this debilitating respiratory condition.

Notably, a study illustrating the inhibitory effect of RWJ-355871 on cath G activity in an acute inflammation model induced by lipopolysaccharide, shedding light on potential therapeutic avenues for modulating the impact of cath G in inflammatory processes within the lung [29].

One intriguing area of research revolves around cath G and its potential role in the aging of the lung. Cath G is a serine protease, primarily known for its involvement in immune responses and inflammatory processes. However, recent scientific investigations have illuminated the possibility that cath G may play a crucial part in the complex interplay of molecular events that underlie the aging of the lungs [30–34].

This is an exploratory study involves a comprehensive examination of the intricate connections between cath G and pulmonary senescence. We aimed to investigate the correlation between cath G upregulation and various cellular processes, including tissue remodeling, and how these processes may influence the aging of the lung and knockout of cath G will be needed in the future for better understanding and trying to stop these pulmonary changes during aging.

The objective of this study was to enhance our comprehension of age-related structural alterations in the rat lung based on lysosomal peptidase profiling, histological, immunohistochemical and ultrastructural parameters.

## 2 Materials and methods

### 2.1. Animals

The study was conducted on male Sprague–Dawley rats aged 4 (average 350 g, n = 10) and 24 months (average 600 g, n = 10). The rats were bred and maintained in Zoology Department Animal Facility, Faculty of Science, Al-Azhar. University under standard laboratory conditions with specific environmental settings at a temperature of 21±2 °C with a humidity of 55±5% and a 12-hour dark/light cycle and provided unrestricted access to food and water. The study was carried out under the guidelines of the Ethics Committee of the Faculty of Science, Al-Azhar University, Assiut, Egypt (AZHAR 8/2022).

### 2.2. Collection of lung tissues

The rats were sacrificed with carbon dioxide asphyxiation and immediately dissected, and lung tissues were dissected and divided into three parts. The first part was fixed in 10% neutral buffered formalin for histological and immunohistochemical work. The second part was kept at—80 °C for proteomic analysis. The third part was fixed in 2.5% glutaraldehyde in phosphate buffer (PH 7.2) for 24 hours for ultrastructural analysis.

### 2.3. Protein extraction

For a comparative proteomic analysis, fifty milligrams of lung tissues (n = 5 per group) were homogenized in 600 μL of a lysis buffer (0.2 M sodium acetate, pH 5.5). To achieve cell disruption, a series of 10-second bursts of sonication using an ultrasonic probe (Bandelin Sonopuls GM 70) were employed on ice. This process was repeated until a uniformly blended mixture was obtained. To promote protein solubilization, 6 μL of 10% Triton X-100 was added. Subsequently, each sample was vortexed three times, followed by 10-minute incubation periods on ice after the first two vortex steps and a 30-minute incubation period after the final vortex. The samples were centrifuged at 12,000 g for 10 minutes at 4 °C. The supernatant was transferred to fresh tubes, while the pellets were discarded.

The determination of protein content was conducted using the BCA protein assay kit (Pierce, Prod. #23225). Subsequently, the samples were aliquoted and stored at −20°C for immunoblotting.

### 2.4. Zymography analysis

Zymography is an SDS-PAGE-based functional assay used to determine enzymatic activities of proteolytic enzymes, particularly metalloproteinases [35]. The methodology described by [36] was adopted for this purpose, focusing on the observation of metalloprotease activity via gelatin digestion within the gel matrix.

20 ug of extracted proteins were mixed with Laemmli sample buffer at a 4:1 ratio and separated on a 10% SDS-PAGE gel without reducing agents. Gelatin at a concentration of 1 mg/mL was included in the gel. For molecular weight determination, pre-stained, low-range SDS-PAGE standards from Bio-Rad were employed. Post-electrophoresis, the gels underwent a 1-hour washing step in 2% Triton X-100 followed by a 48-hour incubation in zymography activation buffer (50 mmol/L Tris–HCl, 5 mmol/L CaCl2 at pH 7.4). A ChemiDoc Universal Hood from Bio-Rad equipped with ultraviolet light scanning was used to evaluate proteolytic activity. The resulting images were subjected to qualitative analysis (densitometry), while the quantification of gelatinase activities was analyzed by ImageJ software (NIH, Bethesda, MD, USA) [12–14].

### 2.5. Active-site labeling of the peptidases and pulldown

The methodology involved the selective extraction of active proteases from protein samples using the Pull-Down technique. Biotinylated protease inhibitors and streptavidin beads facilitated this process, as described by [12–14].

Initially, 200 μL (300 ug) of protein sample was combined with 800 μL of binding buffer (20 Mm sodium acetate, pH 5.5; 150 mM sodium chloride; 0.1% triton X-100; 5 ug/ml E-64; 5 ug/ml leupeptin; and 1 mM PMSF) and centrifuged at 13,000 rpm for five minutes at 4°C using an Eppendorf Centrifuge 5415 R. The obtained supernatant was meticulously transferred to a new reaction tube. Subsequently, 1 μL of each specific protease inhibitor for cysteine (DCG-04 from MedKoo Bioscience, Inc.), aspartic (pepstatin A-biotin from Biosynth, England), and serine proteases (FP-biotin from Santa Cruz Biotechnology, USA) was added individually to the mixture. The mixture was incubated at 37 °C for 30 minutes with continuous shaking. To ensure the Pull-Down process, an additional 80 μL of a 50% streptavidin beads suspension (EMD Millipore Corporation, USA.) was introduced and incubated overnight at 4 °C with rotational agitation.

The next day, samples were centrifuged at 3,000 rpm for five minutes, supernatants were discarded, and beads underwent thorough washing—five washing steps with Wash Buffer (20 mM sodium acetate (Ph 5.5), 150 mM sodium chloride, and 0.1% triton X-100 and twice in 10 mM Tris-HCL, pH 6.8). Subsequently, 40 μL of 2 x Sample Buffer was added to the beads and heated at 95 °C for seven minutes. Eluted proteins were separated on 12.5% acrylamide gel and transferred onto a nitrocellulose membrane. Following that, distinct bands were visualized by employing antibodies that target cathepsins B, L, D, X, and G (Biotechne, USA) at concentrations of 1:1000 in TBST.

### 2.6. Western blot

Proteins (20 μg) were separated on a 12.5% SDS-PAGE gel and transferred to a nitrocellulose membrane (Santa Cruz Biotechnology, Dallas, TX, USA) using a semi-dry blotting system at 25 V for 30 minutes. To mitigate nonspecific binding, membranes were blocked for 3 hours with a solution containing 3% BSA in PBS. Subsequently, membranes were incubated with primary antibodies against cathepsins B, D, X, or G (ThermoFisher Scientific, Waltham, MA, USA) (1:2000 in 3% BSA in TBST). Following this, the membrane underwent five washing steps with TBST before being incubated for another 90 minutes with corresponding secondary antibodies, Goat Anti Mouse IgG (Sigma Aldrish, Germany), at a concentration of 1:40000 in 0.5% BSA in TBST (Sigma Aldrish, Germany). After three washing steps with PBST and one with PBS, membranes were developed with ECL plus Western blotting detection reagent (Amersham, GE Healthcare, Vienna, Austria) and exposed to X-ray films (Amersham Hyper film ECL, GE Healthcare, Vienna, Austria). The experimental films were scanned and analyzed using ImageJ software (NIH, Bethesda, MD, USA) [12–14].

### 2.7. Histopathology

The formalin-fixed tissues were subjected to dehydration in ascending grades of ethyl alcohol, clearing in xylol, embedding in paraplast, and subsequently prepared into paraffin blocks. Paraffin sections of 5 μm thickness were obtained using a Leica microtome and subsequently stained with the following histological stains:

1. Hematoxylin and Eosin stain for general histological examination [37].
2. Picro-Sirius Red stain for detection of collagen fibers [38, 39].

### 2.8. Semithin sections and transmission electron microscopy

The lung specimens (two mm thick) were initially fixed in 2.5% glutaraldehyde in phosphate buffer (pH 7.2) for 24 hours, washed two times in 0.1 M phosphate buffer, and post-fixed in 1% osmium tetraoxide. Following post-fixation, the specimens were dehydrated using ascending alcohol gradients and embedded in araldite resin. Semi-thin sections, 1 μm of thickness, were stained with 1% toluidine blue. Ultrathin sections were produced with a Reichert ultra-microtome and stained with uranyl acetate and lead nitrate. Ultrathin sections were examined, and electron micrographs were captured using a Jeol Jem 1200 EX Transmission Electron Microscope at the Electron Microscope Center of Assiut University.

Specific structures on representative images were highlighted by Adobe Photoshop (ver. 6.0; Adobe Systems) [40, 41]. Paraffin sections and semithin sections were examined using an OLYMPUS BX51 microscope, and images were picked up using an OLYMPUS DP72 camera integrated with the microscope.

### 2.9. Immunohistochemical detection of cathepsin G

Immunohistochemical detection of cath G in paraffin sections was carried out. The paraffin-embedded lung tissues underwent a dewaxing process involving two 10-minute immersions in xylene. Subsequently, the tissues on the slides were rehydrated through sequential immersions in 100%, 90%, 80%, and 70% ethanol, each for 3 minutes, followed by three 5-minute rinses in 0.01 M PBS (pH 7.4). The slides were immersed in 10 mM sodium citrate buffer (pH 6.0) for antigen retrieval and subjected to heating (95–98°C) in a water bath for 20 minutes, followed by a 20-minute cooling period at room temperature. Subsequently, the sections were rinsed in PBS (pH 7.4) three times for 5 minutes each. Using the Dako pen (Agilent Technologies, S2002), a circle was drawn around the tissue and then to inhibit endogenous peroxidase activity, the lung tissues were exposed to 3% hydrogen peroxide for 10 minutes at room temperature, followed by washing in 0.01 M PBS (pH 7.4), three times for 5 minutes each. Sections were then treated with a blocking solution that included 5% donkey serum, 2% bovine serum albumin, and 0.1% Triton X100 for one hour at room temperature. Following this, the sections were incubated with primary antibodies specific to cath G (Cell Signaling Technology 83578; concentration 1:200) for 72 hours at 4°C. The slides were washed with PBS (pH 7.4) four times for 5 minutes each and then subjected to incubation with a secondary antibody (Cell Signalling Technology, Goat antirabbit 7074, conc. 1:400) for 2 hours at room temperature. Subsequently, the slides were rinsed in PBS (pH 7.4) three times for 5 minutes each and incubated with drops of a chromogen diaminobenzidine substrate (DAB, Thermo Fisher Scientific, USA) for 2–3 minutes. The staining reaction was controlled under the microscope. Finally, the slides were rinsed with distilled water to stop the reaction. The sections were counterstained with Harris hematoxylin for 30 seconds. Subsequently, the sections underwent dehydration in a series of graded alcohols (ethanol 70%, 80%, 90%, and 100%), followed by two clearings in xylene. They were then mounted with DPX. Negative controls were incubated without primary antibodies. Immunohistochemical staining was assessed using an OLYMPUS BX51 microscope, and images were captured with an OLYMPUS DP72 camera attached to the microscope [42, 43].

### 2.10. Statistical analysis

The statistical analyses were performed using GraphPad Prism version 6.0c (GraphPad, La Jolla, CA) [26]. Data were analyzed by unpaired Student’s t-test. The probability level of p < 0.05 was considered statistically significant. Data are presented as mean ± SD.

## 3. Results

### 3.1. In vitro active-site labeling and pull-down of cysteine peptidases

**Fig 1:**
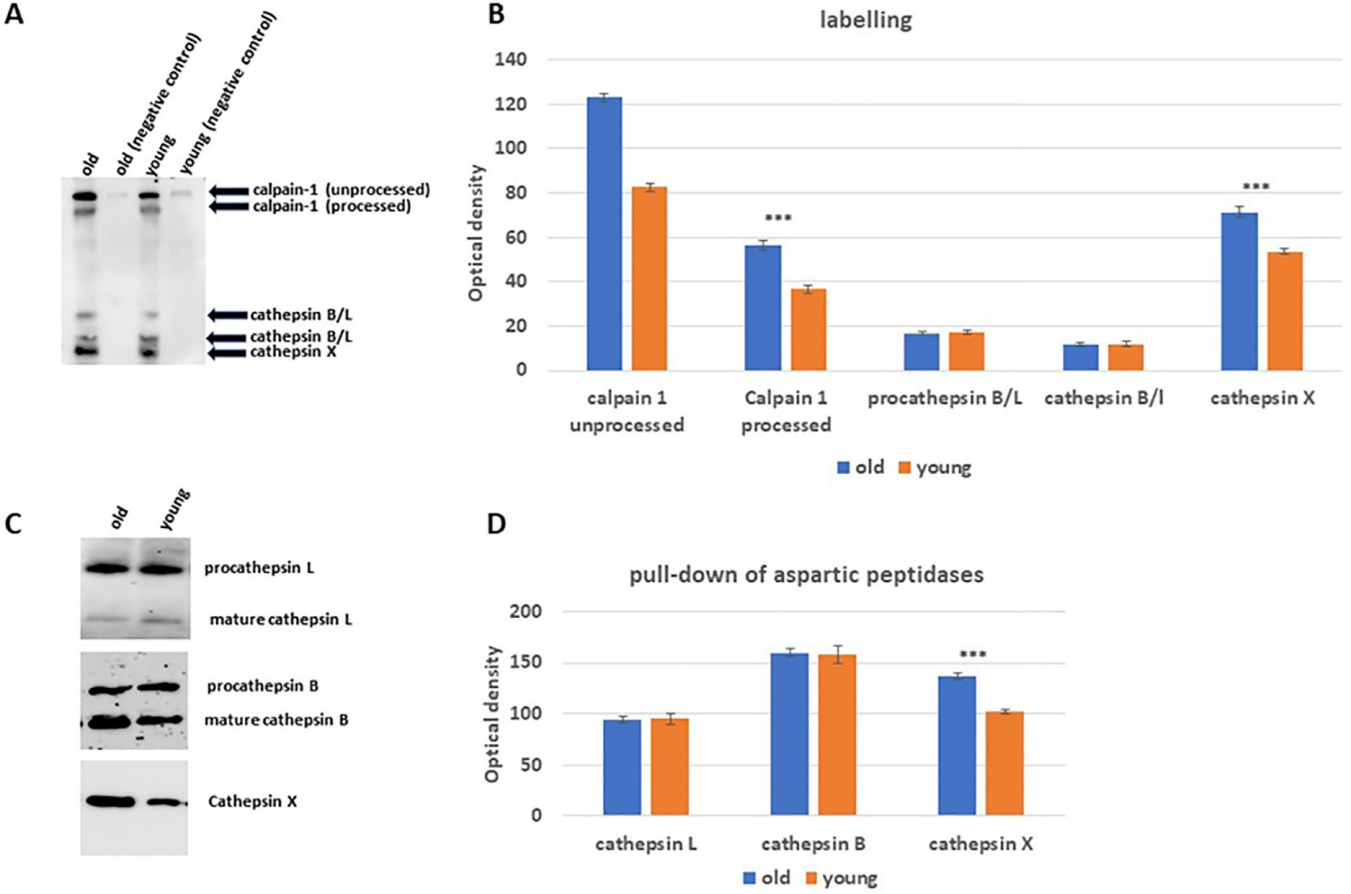
The activity levels of specific cysteine peptidases in lung tissue extracts, previously marked with DCG-04, from both old and young rats. (**A**) The proteins extracted from tissue samples of both old and young rats were labeled using the activity-based probe DCG-04. These labeled proteins were then analyzed through protein electrophoresis and subsequent Western blotting, employing streptavidin-horseradish peroxidase detection. (**B**) The analysis encompassed the assessment of calpain-1 content, encompassing both its unprocessed and processed forms, as well as the unprocessed form of cath B/L, processed form of cath B/L, and cath X bands within lung tissue extracts obtained from both young and old rats. A comparison was made between DCG-04 labeled cells and cells labeled only with DCG-04 (Control). (**C**) The avidin pull-down experiment involving cysteine cathepsins in tissue extracts from both old and young rats was conducted following the outlined procedures in the materials and methods section. Proteins conjugated to avidin Sepharose beads were subsequently analyzed through SDS-PAGE and Western blotting using antibodies specifically targeting human cathepsins B, L, and X. (**D**) The comparative analysis of cellular contents ratio for cathepsins B, L, and X in tissue extracts from both old and young rats. The dataset underwent statistical analysis via two-way ANOVA with subsequent Dunnett’s post hoc analysis (*** p < 0.001; n = 5). Statistical computations were performed using GraphPad Prism.

This investigation was conducted to determine the extent to which the active sites of cysteine cathepsins can be accessed by the activity-based probe DCG04. Initially, DCG04 was designed to be a specific activity-based probe for targeting cysteine peptidases. In this experiment, protein extracts of both old and young rats (~ 30 μg) were incubated with 10 μM DCG04 for 30 minutes. The DCG04-labelled proteins were then pulled down using streptavidin A agarose beads. The agarose beads bound with proteins were subsequently subjected to elution using a protein sample buffer. The eluted proteins were then subjected to western blotting analysis, employing streptavidin-horseradish peroxidases and immunodetection using specific antibodies against the well-known active cysteine cathepsins, including cathepsins B, L, X, K, and S. To serve as a negative control, unlabeled DCG04 (E64) tissues were utilized in the experiment. Upon analysis, a band with a molecular weight of 30 kDa, corresponding to cath X, was observed to migrate at the same level. This band exhibited higher expression (P value = 0.0016) levels in old lung tissues compared to young lung tissues. Additionally, bands at 33 and 42 kDa, likely representing both immature and mature forms of cath L and B, respectively, were detected. However, no significant differences in band intensities were observed between old and young lung tissues. Furthermore, two additional bands at 68 and 80 kDa, corresponding to the unprocessed and processed forms of calpain-1, were identified in both tissue types. Notably, both forms were found to be more highly expressed in old tissues, approximately 1.5 times greater than in young tissues (Fig 1A). The immunodetection experiments utilizing antibodies specific to cathepsins B, L and X corroborated the previous findings. No differences in the expression levels of cath B or Lwere observed between old and young lung tissues. However, cath X exhibited significantly higher expression levels (approximately 1.3 times greater, P value equals 0.0016) in old tissues (Fig 1B).

### 3.2. In vitro labelling and pull-down of aspartic peptidases

**Fig 2:**
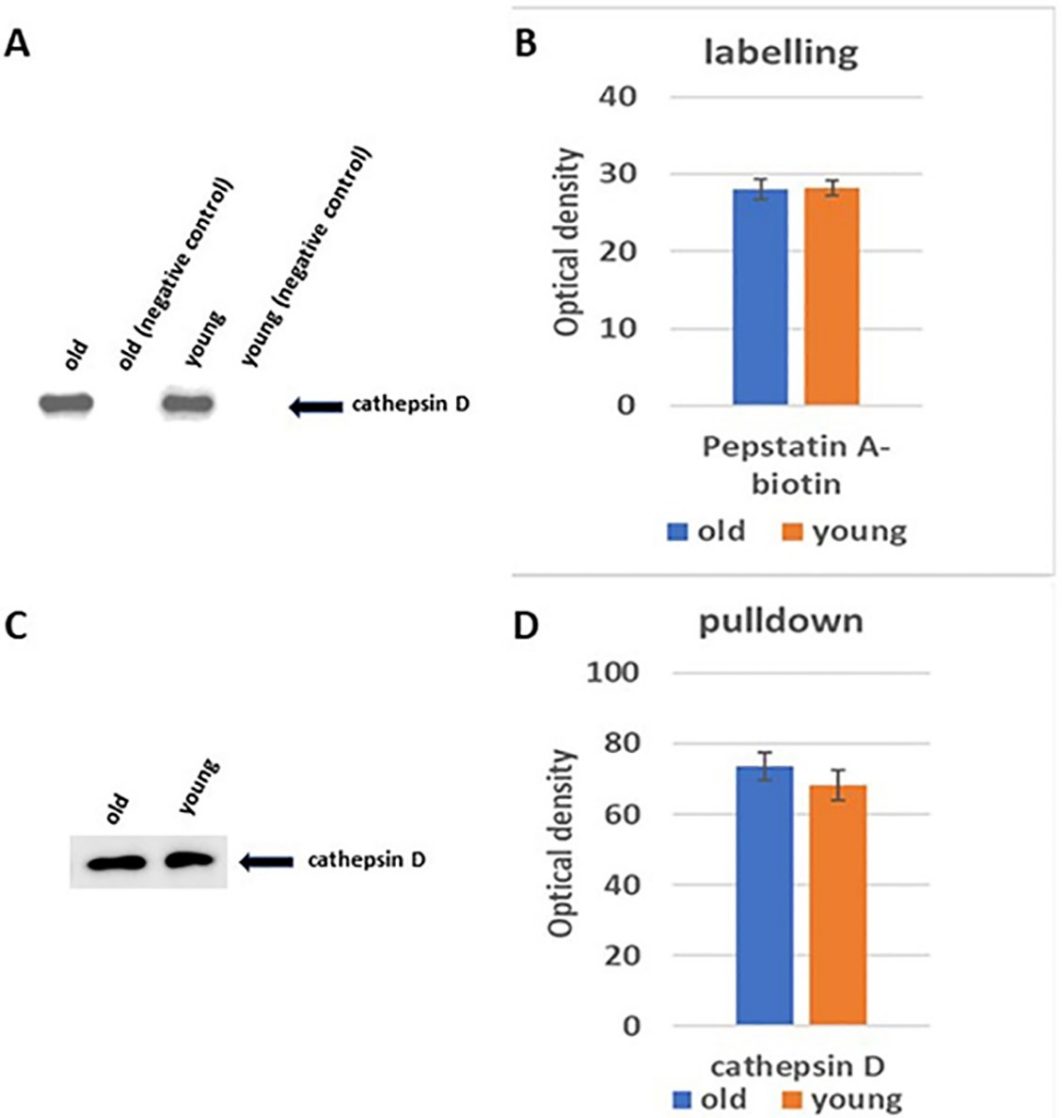
Displays the activity levels of specific aspartic peptidases in lung tissue extracts previously labeled with pepstatin A-biotin, obtained from both old and young rats. (**A**) proteins from lung tissue samples of both old and young groups were labeled using pepstatin A-biotin and subjected to protein electrophoresis and subsequent western blotting, detected via streptavidin-horseradish peroxidase. (**B**) Focuses on assessing cath D bands within these lung tissue extracts, comparing the pepstatin-A labeled aspartic peptidases between old and young rats. (**C**) Illustrates the avidin pull-down experiment targeting aspartic cathepsins in tissue extracts from both old and young groups, followed by SDS-PAGE and Western blotting using antibodies specific to human cathepsins D. Lastly, (**D**) Presents the comparative analysis of tissue content ratios for cath D in both old and young rat extracts, analyzed via two-way ANOVA with Dunnett’s post hoc analysis (n = 5) using GraphPad Prism for statistical computations.

In order to investigate the proteolytic activities exhibited by aspartic peptidases, 30 μg of lung protein extracts from young and old rats were subjected to an incubation process with the activity-based probe pepstatin A-biotin. Subsequently, the proteins labeled by the aspartic peptidases were selectively isolated by employing streptavidin A agarose beads. These beads, which had bound with the targeted proteins, were then subjected to elution using a protein sample buffer. The eluted proteins were subsequently subjected to western blotting and immunodetection using specific antibodies to cathepsins D and E. Wherein streptavidin-horseradish peroxidases were employed for visualizing the active aspartic cathepsins, including cathepsins D and E. As a control for comparison, unperturbed tissues treated with unlabeled pepstatin A were included in the experimental design. A band was a molecular weight 48 kDa corresponds to the full-length form of cath D was detected after streptavidin A in both old and young lung tissues (Fig 2A). The immunodetection analysis of the pulled-down cath D yielded consistent outcomes (Fig 2B). Notably, cath E was not detectable in the experiment.

### 3.3. In vitro labelling and pull-down of serine peptidases

To investigate the proteolytic activities of serine peptidases, we conducted an experiment using lung protein extracts from both young and old rats. Approximately 30 μg of protein extracts were incubated with an activity-based probe called FP-biotin. This probe specifically binds to the serine peptidases. The bounded serine peptidases were then isolated by utilizing streptavidin A agarose beads. These beads selectively bind to the biotin-bounded proteins. To release the bound proteins, the beads were subjected to elution using a protein sample buffer. The eluted proteins were further analyzed using western blotting and immunodetection techniques. Specifically, specific antibodies for cathepsins A and G were used to identify the presence of these proteins.

**Fig 3:**
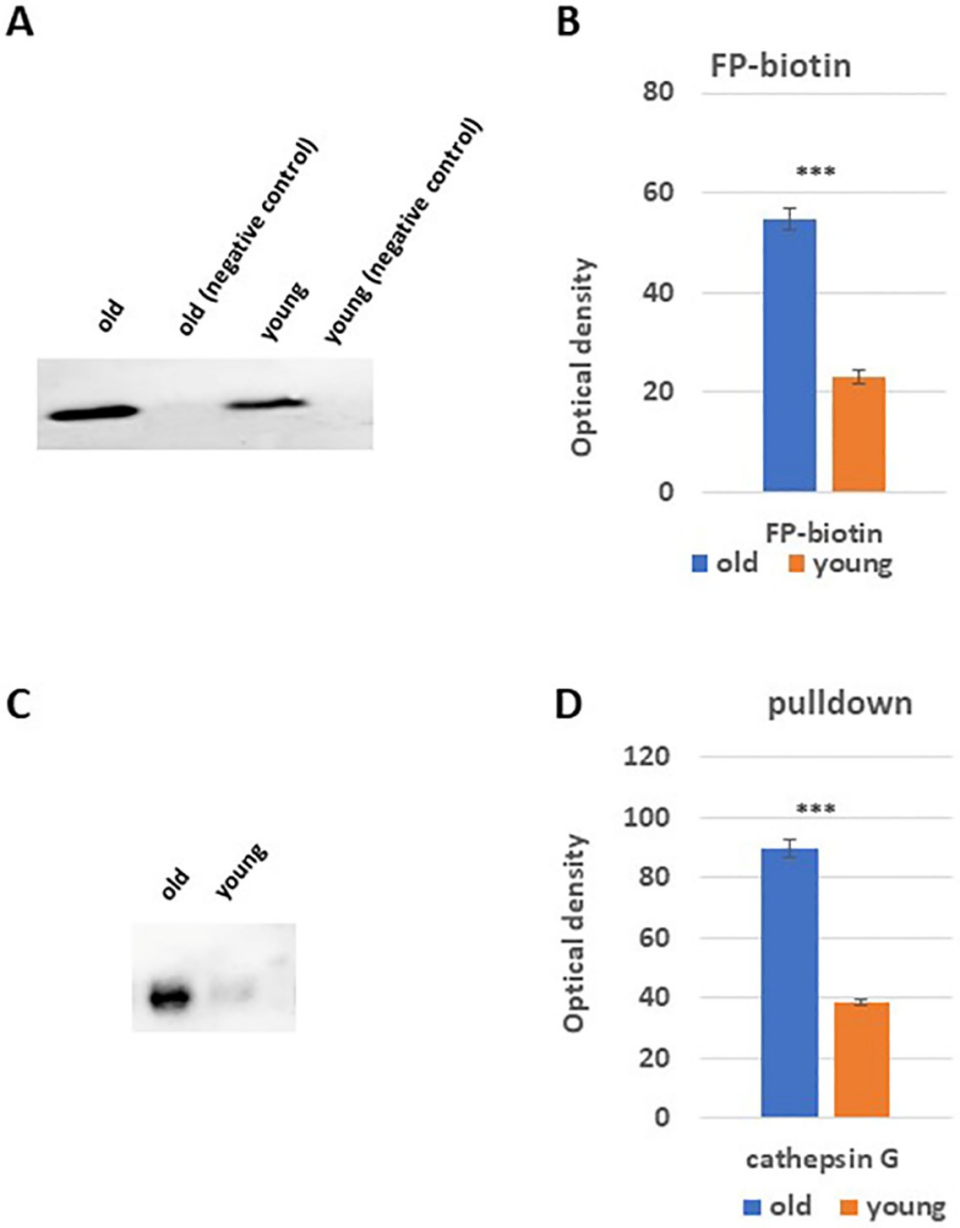
Displays the activity levels of specific serine peptidases in lung tissue extracts labeled with FP-biotin from both old and young rats. (**A**) Proteins from lung tissue samples of both age groups were labeled using FP-biotin, subjected to protein electrophoresis, and analyzed via western blotting using streptavidin-horseradish peroxidase. (**B**) Focuses on comparing the cath G bands in these lung tissue extracts, specifically assessing FP-biotin labeled serine peptidases between old and young rats. (**C**) Illustrates the avidin pull-down experiment targeting serine cathepsins in tissue extracts from both age groups, followed by SDS-PAGE and Western blotting using antibodies specific to human cath G. Lastly, (**D**) Presents a comparative analysis of tissue content ratios for cath G in both old and young rat extracts, analyzed using two-way ANOVA with Dunnett’s post hoc analysis (*** p < 0.001; n = 5) via GraphPad Prism for statistical computations.

In both old and young lung tissues, a band corresponding to the full-length form of cath G with a molecular weight of 28 kDa was observed after FP-biotin labeling (Fig 3A). The old tissues showed significant higher expression level of cath G (P value is less than 0.0001) compared to young tissues (approximately 4.3 times greater). The analysis of the pulled-down cath G using immunodetection showed similar results (Fig 3B). It is important to note that cath A was not detected in the experiment.

### 3.4. Metalloproteinases activities

To assess and compare the activities of metalloproteinases in lung tissues from both young and old subjects, we conducted a gelatin zymography technique.

**Fig 4:**
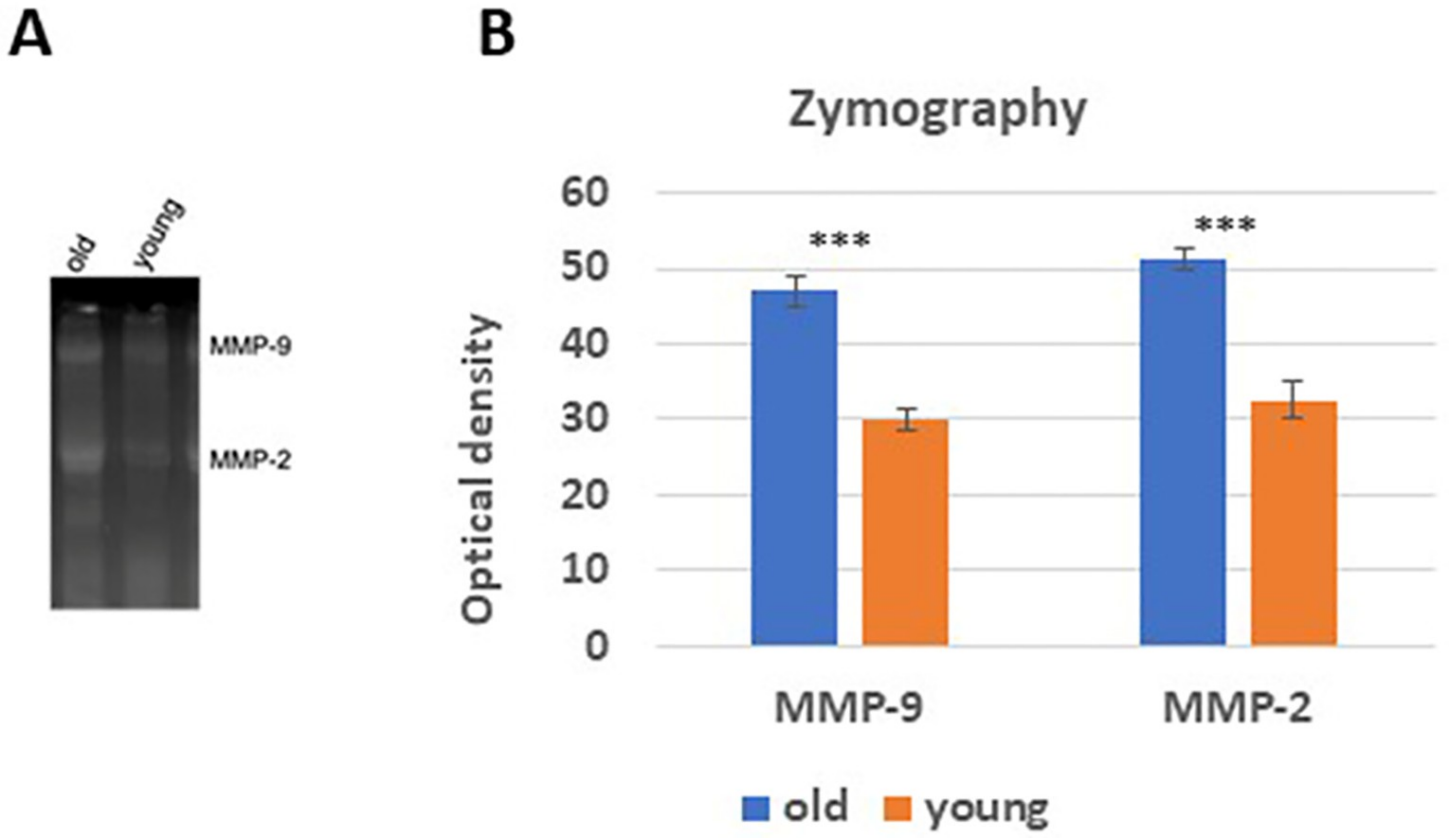
MMP-2 and MMP-9 activities in the lung tissues of both old and young rats. (**A**) Gelatin zymography of MMP-2 and MMP-9 activities in old and young lung tissues. (**B**) Presents a comparative analysis of tissue activity ratios for MMP-2 and MMP-9 in both old and young rat extracts, analyzed using two-way ANOVA with Dunnett’s post hoc analysis (*** p < 0.001; n = 5) via GraphPad Prism for statistical computations.

The gelatin zymography revealed the presence of two prominent bands in the electrophoretic pattern, one at approximately 92 kDa, which correspond to migration pattern of matrix metalloproteinase 9 (MMP-9), and the other at around 72 kDa, corresponding to the migration pattern of matrix metalloproteinase 2 (MMP-2) (Fig 4A).

Our findings indicated significant differences in the activity levels of these metalloproteinases between the young and old subjects. Specifically, the activity of MMP-9 was found to be 40% lower in the lung tissues of the young rats when compared to their older counterparts. Additionally, the activity of MMP-2 exhibited a reduction of approximately 34% in the lung tissues of the young rats compared to the older group (Fig 4B).

### 3.5. Fibronectin fragmentation

Building on previous findings [15, 16] indicating the upregulation of certain cathepsins in aged tissues, potentially leading to the degradation of extracellular matrix (ECM) components such as fibronectin, we deemed it valuable to compare the extent of fibronectin degradation in both aged and youthful lung tissues. To explore the potential degradation or fragmentation of fibronectin during the aging process, we designed an experiment involving both young and old rat lung tissues. Our methodology included subjecting these tissues to protein electrophoresis under reducing conditions, followed by the separation of proteins, transfer to a nitrocellulose membrane, and subsequent treatment with antibodies specifically targeting fibronectin.

**Fig 5:**
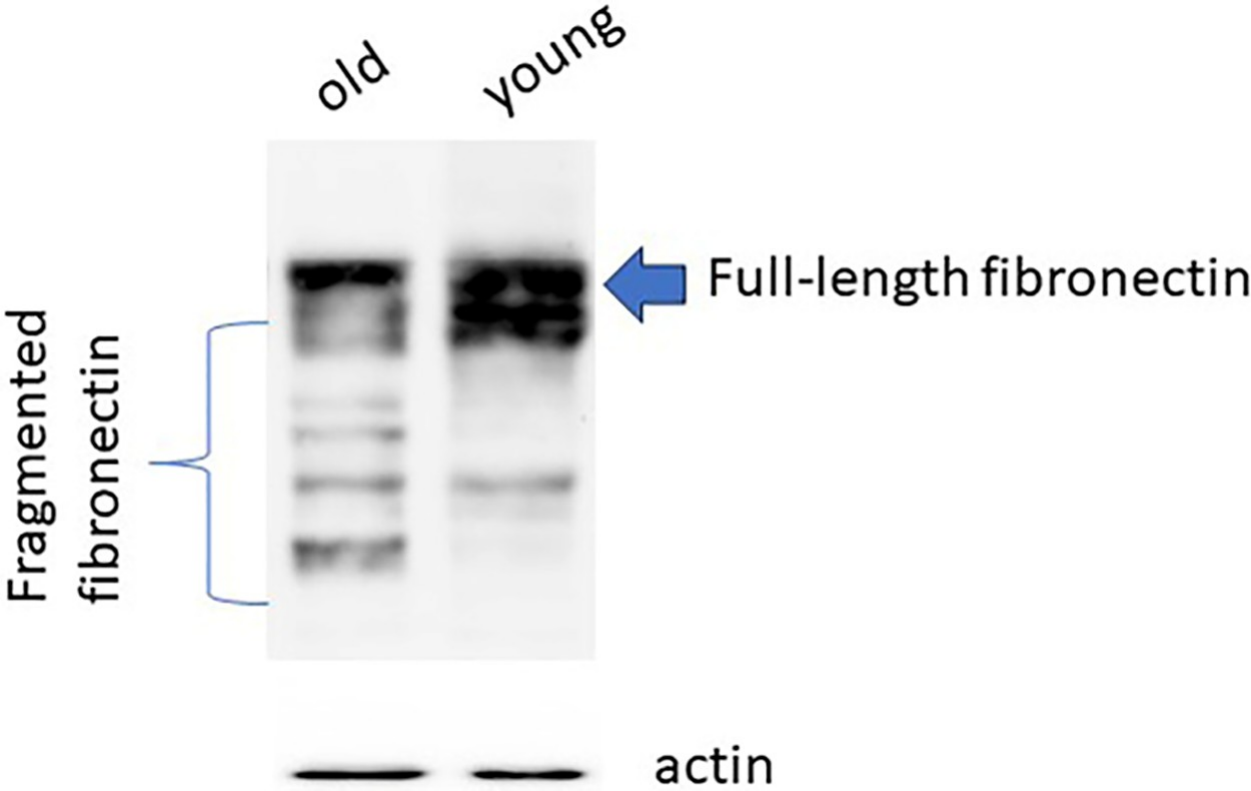
Fibronectin degradation analysis. 20 μg lung tissue proteins of both old and young rats were exposed to SDS-PAGE and Western blotting using antibodies specific to human fibronectin. This experiment was performed five times with similar results.

Distinct single and smaller weak bands were observed in the case of young rats. However, in the old rats more than five bands became apparent. Notably, the full-length fibronectin band in the old rat tissues exhibited a significantly lower intensity compared to the corresponding band in the young rat tissues. This discrepancy strongly suggests that fibronectin undergoes fragmentation in old lung tissues (Fig 5).

### 3.6. Histological and immunohistochmecal investigation

**Fig 6:**
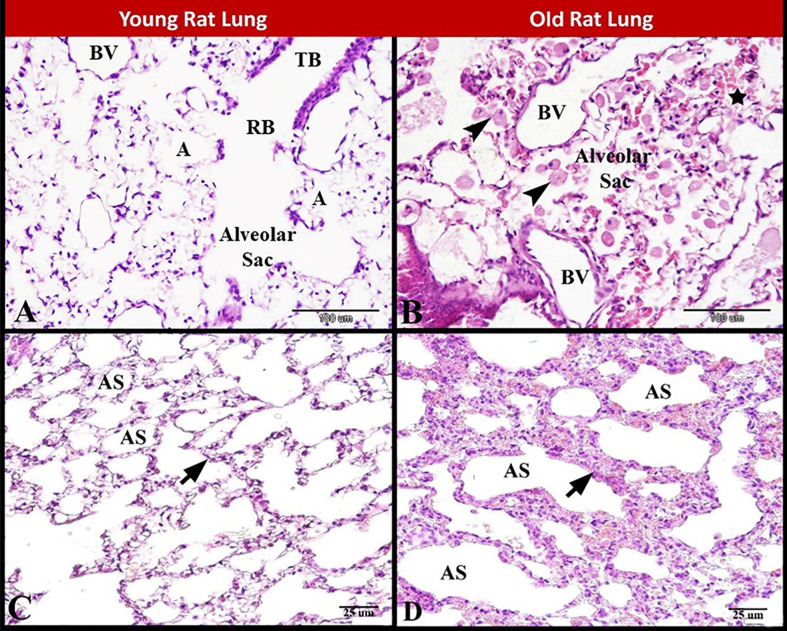
Photomicrograph of paraffin sections showing structural changes during ratpulmonary senescence. **(A**) Young rat lung showing normal terminal bronchiole (TB), respiratory bronchiole (RB), alveolar sac, alveoli (A) and blood vessels (BV). **(B)** Old rat lung showing pulmonary tissue destruction (star), aged blood vessels (BV) and disturbed alveolar sac filled with alveolar macrophages (arrow heads). **(C**) Young rat lung showing normal, small, numerous and evenly distributed air spaces (AS) with normal interstitial tissue (arrow). (**D)** Old rat lung showing few large irregular air spaces (AS) with thickening and abundant interstitial tissue (arrow). Stain: H and E, Scale bar in A & B = 100 μm, Scale bar in C & D = 25 μm.

Microscopical examination revealed that the lungs of young rats exhibited normal terminal bronchiole, respiratory bronchiole, alveolar sac, alveoli and blood vessels (Fig 6A). While the lung of old rats exhibited pulmonary tissue destruction, aged blood vessels and disturbed alveolar sac filled with alveolar macrophages (Fig 6B). The air spaces in young rat lung were normal, small, numerous and evenly distributed with normal amount of the interstitial tissue (Fig 6C). In contrast, in the old rat lung there were few large irregular air spaces with thickening and abundant interstitial tissue (Fig 6D).

**Fig 7:**
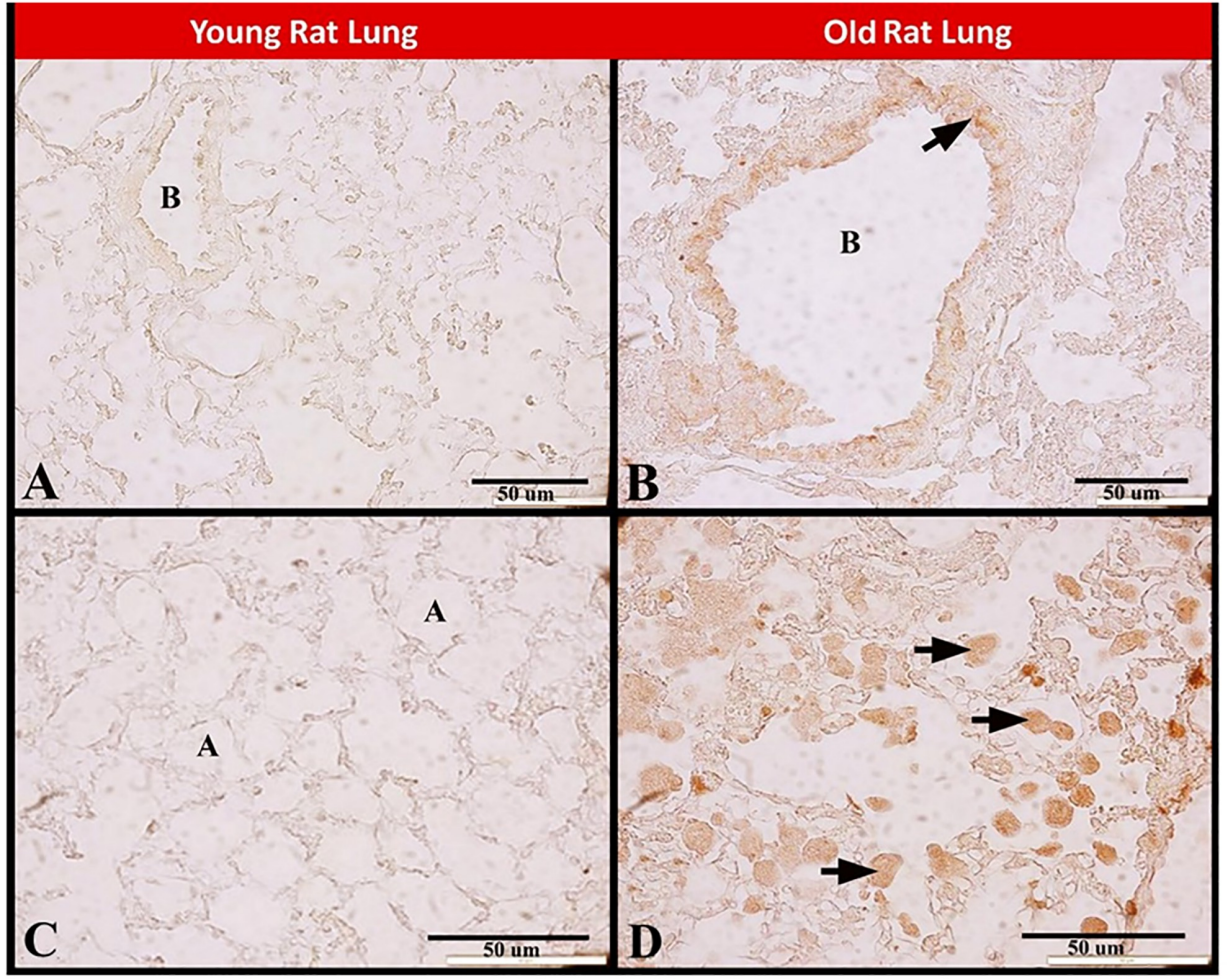
Photomicrograph of paraffin sections showing immunoexpression of cath G during rat pulmonary senescence. **(A, C)** Young rat lung showing no or slight cath G immunostaining in pulmonary bronchioles in **A** and pulmonary alveoli in **C**. **(B, D)** Old rat lung showing abundant strong cath G immunostaining in pulmonary bronchioles (arrow) in **B** and in alveolar macrophages (arrow) in **D**. Pulmonary bronchioles (B) and pulmonary alveoli (A). Scale bar in A-D = 50 μm.

Immunoexpression of cath G showed no or slight cath G immunostaining in pulmonary bronchioles and pulmonary alveoli in young rat lung. Whereas the old rat lung expressed abundant strong cath G immunostaining in pulmonary bronchioles and pulmonary macrophages (Fig 7A–7D).

### 3.7 Ultrastructural investigation

**Fig 8:**
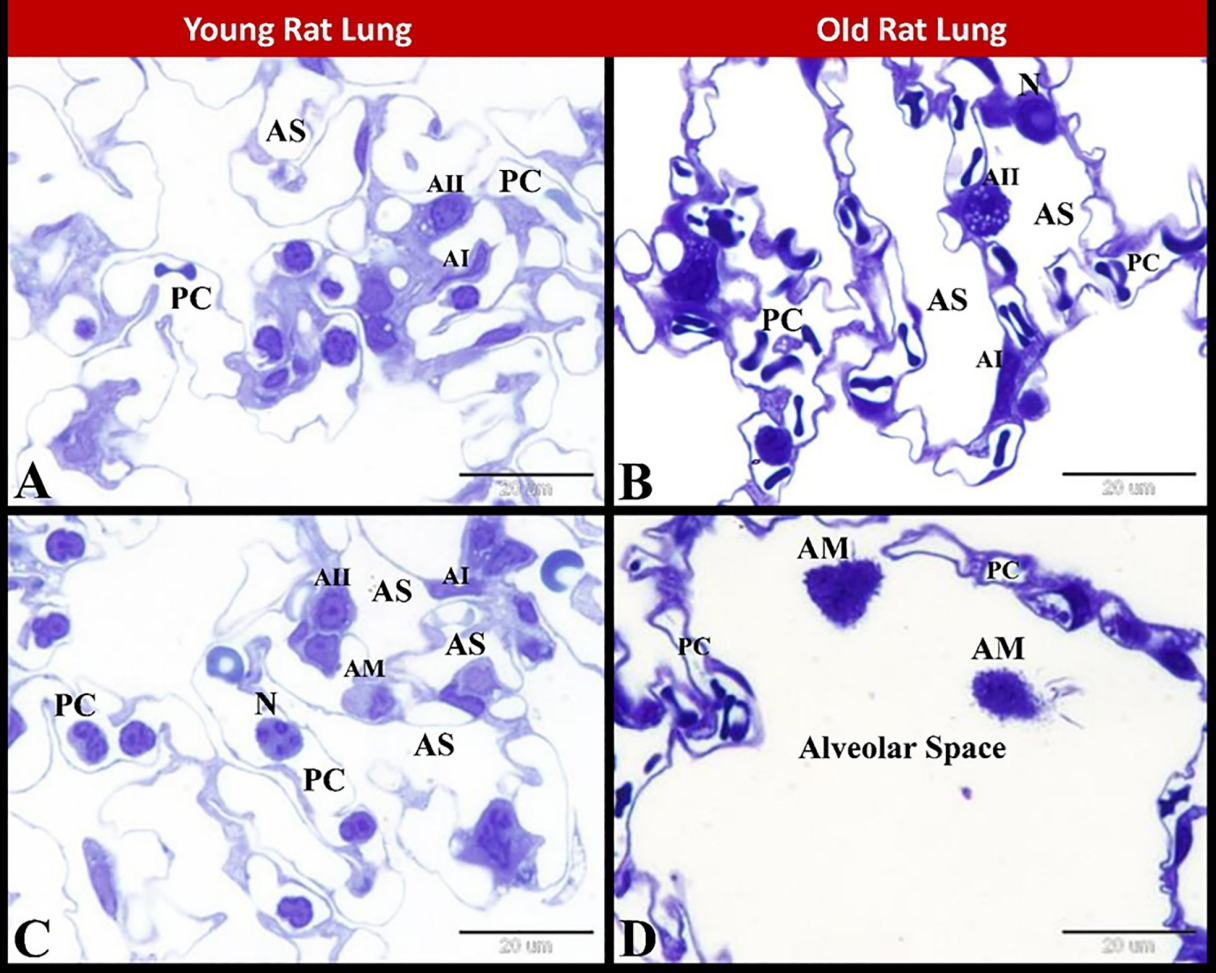
Photomicrograph of semi-thin sections showing structural changes during rat pulmonary senescence. **(A, C)** Young rat lung showing normal small alveolar space, alveolar type 1 and alveolar type 2 cells and normal slightly wide pulmonary capillaries with leucocytes as neutrophils. Note the alveolar macrophage in **C**. **(B, D)** Old rat lung showing in **B** widening of alveolar space, aged alveolar type 1 and alveolar type 2 cells, aged narrow pulmonary capillaries and extra capillary neutrophil in the alveolar space. in **D** showing large wide alveolar space contained aged alveolar macrophage and surrounded by aged narrow pulmonary capillaries. Alveolar space (AS), alveolar type 1 (AI), alveolar type 2 (AII), pulmonary capillaries (PC), neutrophils (N) and alveolar macrophage (AM). Stain: Toluidine blue, Scale bar in A-D = 20 μm.

Examination of the semi-thin sections stained by toluidine blue revealed that the young rat lung had normal small alveolar space, alveolar type 1 and alveolar type 2 cells and normal slightly wide pulmonary capillaries with leucocytes as neutrophils and alveolar macrophages. While the old rat lung showed large wide alveolar space, aged alveolar type 1 and alveolar type 2 cells and aged narrow pulmonary capillaries with extra capillary neutrophils in the alveolar space and aged alveolar macrophage (Fig 8A–8D).

**Fig 9:**
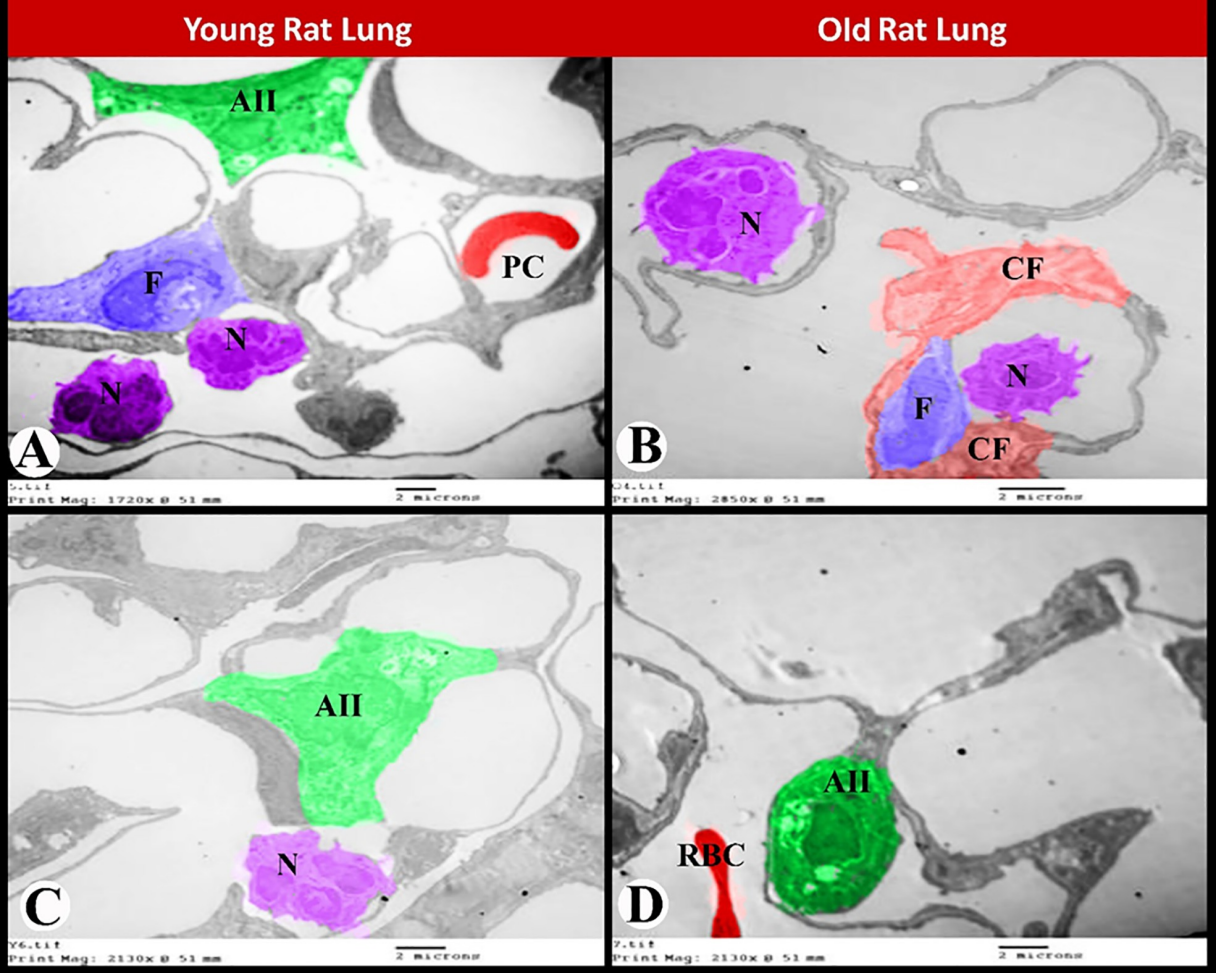
Transmission electron micrograph of ultrathin sections showing ultrastructure changes during rat pulmonary senescence. **(A, C)** Young rat lung showing normal alveolar type 2 cells and normal slightly wide pulmonary capillaries, interstitial fibroblasts and normal neutrophils with its characteristic segmented nucleus and granules. **(B, D)** Old rat lung showing aged neutrophils with its characteristic large size and numerous pseudopodia, interstitial fibroblasts surrounded by abundant collagen fibers, aged alveolar type 2 cells and extravasated red blood cells inside the alveolar space. Alveolar type 2 (AII), pulmonary capillaries (PC), neutrophils (N), fibroblasts (F), collagen fibers (CF) and red blood cells (RBC). Scale bar in A-D = 2 μm.

Transmission electron microscopy showed that the young rat lung had normal alveolar type 2 cells, interstitial fibroblasts and normal neutrophils with its characteristic segmented nucleus and granules. While old rat lung showed aged neutrophils with its characteristic large size and numerous pseudopodia, interstitial fibroblasts surrounded by abundant collagen fibers, aged alveolar type 2 cells and extravasated red blood cells inside the alveolar space (Fig 9A–9D).

## 4. Discussion

Aging is a complex biological process characterized by the progressive decline of physiological functions and an increased susceptibility to age-related diseases. Pulmonary senescence is intricately related to the development of cancer, fibrosis, and inflammation (flogosis). Age-related changes in lung structure and function increase the risk of non-small cell lung cancer (NSCLC), with older patients exhibiting higher postoperative mortality rates. Additionally, Pulmonary senescence contributes to pulmonary fibrosis and chronic inflammation, further exacerbating respiratory health issues [27]. Various molecular mechanisms contribute to aging, including altered protein homeostasis and extracellular matrix (ECM) remodeling. The current study aimed to investigate the relationship between several key molecules, namely cath G, metalloproteinases, cath X, and fibronectin, and their potential role in the aging process.

It has been suggested that increased activity of the lysosomal serine protease cath G may contribute to age-related changes in tissue homeostasis and inflammatory responses [15]. We detected increased protein levels of cath G in the lung tissue of old rats. The overexpression of cath G may lead to the degradation of key ECM components, such as fibronectin, and activation of pro-inflammatory pathways, contributing to tissue dysfunction and aging [15, 16].

Metalloproteinases, a family of enzymes involved in ECM remodeling, are overexpressed in old rats [44]. Metalloproteinases, such as matrix metalloproteinases (MMPs), play a crucial role in maintaining the balance between ECM synthesis and degradation. However, their dysregulation in aging can lead to excessive ECM degradation and impaired tissue integrity. The upregulation of metalloproteinases in old rats may be associated with age-related tissue damage and the accumulation of ECM fragments [45]. In our study the MMP-2 and MMP-9 activities were significantly higher in old lung rat tissues as compared to young ones.

Cath X, another member of the cath family, has been observed to be slightly upregulated in old rats. Cath X, a cysteine carboxypeptidase, plays a significant role in degenerative processes both in normal aging and in various pathological states. Cath X has been implicated in various physiological and pathological processes, including ECM remodeling. Although the specific role of cath X in aging is not well-defined, its upregulation suggests a potential contribution to ECM degradation and tissue remodeling during the aging process [46].

Fibronectin, a major component of the ECM, plays a critical role in maintaining tissue structure, cell adhesion, and migration. In old rats, Fibronectin has been found to be degraded in the lung tissue of old rats, which may be attributed to the increased activity of cath G and other proteases. The degradation of Fibronectin compromises tissue integrity, impairs cell signaling, and disrupts the balance between ECM synthesis and degradation, thus contributing to the aging process [16].

Integrated perspective: The relationship between cath G, metalloproteinases, cath X, and fibronectin provides an integrated perspective on their potential roles in inducing aging. Upregulation of cath G and metalloproteinases in old rats indicates increased proteolytic activity, leading to the degradation of key ECM components, including fibronectin. This ECM remodeling disrupts tissue structure and function, contributing to age-related tissue dysfunction and inflammation. The slight upregulation of cath X may further contribute to ECM degradation, although its specific role in aging requires further investigation.

Pulmonary senescence is associated with various morphological, molecular, and functional changes in the intrapulmonary bronchi, bronchioles, alveoli and pulmonary vascular system in addition to alterations in elastic and collagen fibers. This occurs as a result of accumulation of a wide variety of molecular and cellular damage over time and accompanied with immune and oxidative imbalance. Herein, we highlighted the molecular and cellular aspects of pulmonary aging, and how this predisposes to the pathogenesis of pulmonary disease such as COVID-19 pandemic. Lungs are composed of unique panoply of cell types that face ongoing chemical, mechanical, biological, immunological, and xenobiotic stress over a lifetime [47].

It was proposed that there are nine hallmarks of aging, i.e. genomic instability, telomere attrition, epigenetic alterations, loss of proteostasis, deregulated nutrient sensing, mitochondrial dysfunction, cellular senescence, altered intercellular communication and stem cell exhaustion [48, 49].

In the current study the lung of old rats exhibited pulmonary tissue destruction, aged blood vessels and disturbed alveolar sacs filled with foamy alveolar macrophages. Herein we found in the old rat lung there were few large irregular air spaces with thickening and abundant interstitial tissue. We also found increased peribronchiolar and perivascular collagen fibers distribution in old rat lung compared to young rat lung. Aging of neutrophils and alveolar macrophages and changes in extracellular collagen fibers demonstrate how innate and adaptive immunity within the lung are altered with age [47]. Natural pulmonary senescence is associated with structural, molecular, and physiological changes that cause alterations in lung function, decrease pulmonary remodeling and regenerative capacity, and increased susceptibility to acute and chronic lung diseases. As the pulmonary senescence lead to alterations in cellular function and cell-to-cell interactions of pulmonary resident cells and systemic immune cells which contribute to a higher risk of increased susceptibility to infection and facilitate the development and progression of disease [50]. Alveolar senescence is an irreversible cell-cycle arrest that has a crucial role in pulmonary senescence and show very distinctive changes in morphology with accumulation of transcriptionally inactive heterochromatic structure [51]. In the present work, examination of the semi-thin sections stained by toluidine blue revealed that the old rat lung showed large wide alveolar space, aged alveolar type 1 and alveolar type 2 cells and aged narrow pulmonary capillaries with extra capillary neutrophil in the alveolar space and aged alveolar macrophage.

With aging, we found increased numbers of alveolar macrophages, cells essential for lung homeostasis. Furthermore, aging vastly down-regulates cell cycling pathways in alveolar macrophages and impairs the ability of alveolar macrophages to limit lung destruction. Moreover, aging decreases alveolar macrophage phagocytosis of apoptotic cells. Thus, aging induces defective phagocytosis by alveolar macrophages and increases lung damage. This indicated that therapies promoting the function of alveolar macrophages may improve outcomes in older people infected with respiratory viruses [52]. It has recently been hypothesized that increased cell apoptosis as a result of oxidative stress may have a central role in aging [53]. It was found that the alveolar size declined significantly in old mice concomitant with a widening of alveolar ducts and late alveolarization. Despite age-related lung remodeling, interestingly the number of alveolar type II cells per alveolus showed a tightly controlled relation with increased age [54].

Mammalian aging is accompanied by increased levels of oxidative damage of DNA, proteins, and lipids as a result of unbalanced prooxidant and antioxidant activities. Several accumulating evidence indicated that oxidative imbalance is a major physiological inducer of aging [55].

The old rat lung expressed abundant strong cath G immunostaining in the epithelial cells of the pulmonary bronchioles and pulmonary macrophages. It was suggested that the phospholipid transfer protein (PLTP) was a protective factor and prevents pulmonary inflammation and destruction. Whereas cath G had proteolytic cleavage to PLTP and may enhance the injurious pulmonary inflammatory responses [33]. It was suggested that in brain of old rats, the increased levels of cathepsins D, E, and B and the decrease in cath L activity were related to both the neuronal degeneration and the reactivation of glial cells during the normal aging process of the brain [56].

The lung extracellular matrix contained many elastic fibers and few collagenous fibers which secreted by fibroblasts. Aging or destruction of theses pulmonary fibroblasts is vulnerable to pathological structural changes upon upregulation of serine proteases, including cath G [57]. So we suggested that aging is characterized by high levels of protease activity leading to degradation of elastin followed by loss of elasticity of the lung and the development of emphysema. Elastin is an important structural component of the lung parenchyma as it supports the expansion and recoil of the alveoli during breathing.

Transmission electron microscopy in the present study showed that the old rat lung has aged neutrophils with characteristic large size and numerous pseudopodia, interstitial fibroblasts surrounded by abundant collagen fibers, aged alveolar type 2 cells and extravasated red blood cells inside the alveolar space. Aged neutrophils had impaired superoxide generation and phagocytic activity [58]. Pulmonary macrophages have the ability to phagocytosis of aging neutrophils and the programmed cell death in the neutrophil leads to its recognition by macrophages [59].

Muco-obstructive lung diseases are characterized by extensive bronchiectasis due to the uncontrolled release of neutrophil serine proteases into the airways especially cath G [34]. Aging and chronic inflammation produce complex structural and biochemical alterations to the lung which affect breathing. Mice deficient in surfactant protein D showed progressive age-related lung damages characterized by tissue destruction/remodeling, accumulation of foamy macrophages and change in surfactant composition [60]. Senescence and or apoptosis of alveolar type II pneumocyte drives progressive pulmonary fibrosis [61–63].

Aging is associated with deteriorating health, including the escalating risk of diseases and a diminished ability to repair following injury and is associated with immune dysfunction including myeloid cell immunosenescence. Immunosenescence is accompanied by low-grade chronic inflammageing and increased levels of circulating pro-inflammatory cytokines such as tumor necrosis factor (TNF), interleukin (IL)-1β and IL-6. However, in healthy aging, there is a production of anti-inflammatory cytokines such as transforming growth factor-β1 (TGF-β1) and IL-10, which may overcompensate the pro-inflammatory state. Imbalanced pro- and anti-inflammatory factors during aging have a significant influence on macrophage function and further impact the severity of age-related diseases. They play a crucial role in maintaining homeostasis as they can remove deleterious senescent cells that increase during aging. They can acquire many functional states ranging from pro-inflammatory, anti-tumorigenic to anti-inflammatory, pro-tumorigenic or wound healing macrophages [64]. The importance of investigation of pulmonary senescence that it is considered as a critical factor in the development of certain pathologies, significantly influencing outcomes in non-small cell lung cancer (NSCLC). For example, nearly half of NSCLC patients are diagnosed at 70 years or older, and the age-related impact is evident as the postoperative mortality rate rises dramatically from 1.7% in patients under 60 to 9.4% in octogenarians [65]. In summary, upregulation of cathepsin G and X in the lungs can contribute to lung senescence by promoting inflammation and degradation of extracellular matrix components, leading to tissue damage and impaired repair mechanisms. These proteases can also induce cellular stress and apoptosis, accelerating the aging process of lung cells. Consequently, this upregulation may exacerbate age-related pulmonary diseases, reducing overall lung function and resilience [14, 46].

## 5. Conclusion

Our results showed that the expression levels of cathepsins G and X were notably higher in old rat lung tissues compared to young rat lung tissues. Zymography analysis revealed elevated MMP activity in the old lung tissues, with significant degradation of fibronectin, an essential ECM component. Numerous histological and ultrastructural alterations were observed in old lung tissues compared to young lung tissues. We conclude that the upregulation of cathepsin G, metalloproteinases, cathepsin X, and the degradation of fibronectin in old rats provide insights into the molecular mechanisms underlying Pulmonary senescence. Cathepsin G has an important role in aging lung biology; these observations offer the promise of deeper insights into the aging process and potentially novel approaches to mitigate the lung changes associated with aging, increase resistance to infectious diseases, and promote healthier respiratory aging.

## Supporting information

S1 File This file includes the raw statistical data tables and the original Western blot images for all the different experiments conducted in the study. (DOCX)
