# Supplementary material for: Age-related lung changes linked to altered lysosomal protease profile, histology, and ultrastructure
Source: PLoS One. 2024 Dec 20;19(12):e0311760. doi: 10.1371/journal.pone.0311760 (PMC11661583; doi:10.1371/journal.pone.0311760)
Supplement: S1 File — (DOCX) [file pone.0311760.s001.docx]

**Supplementary Data**

**Fig. 1 Labelling and pulldown of cysteine cathepsins (B,L and X)**


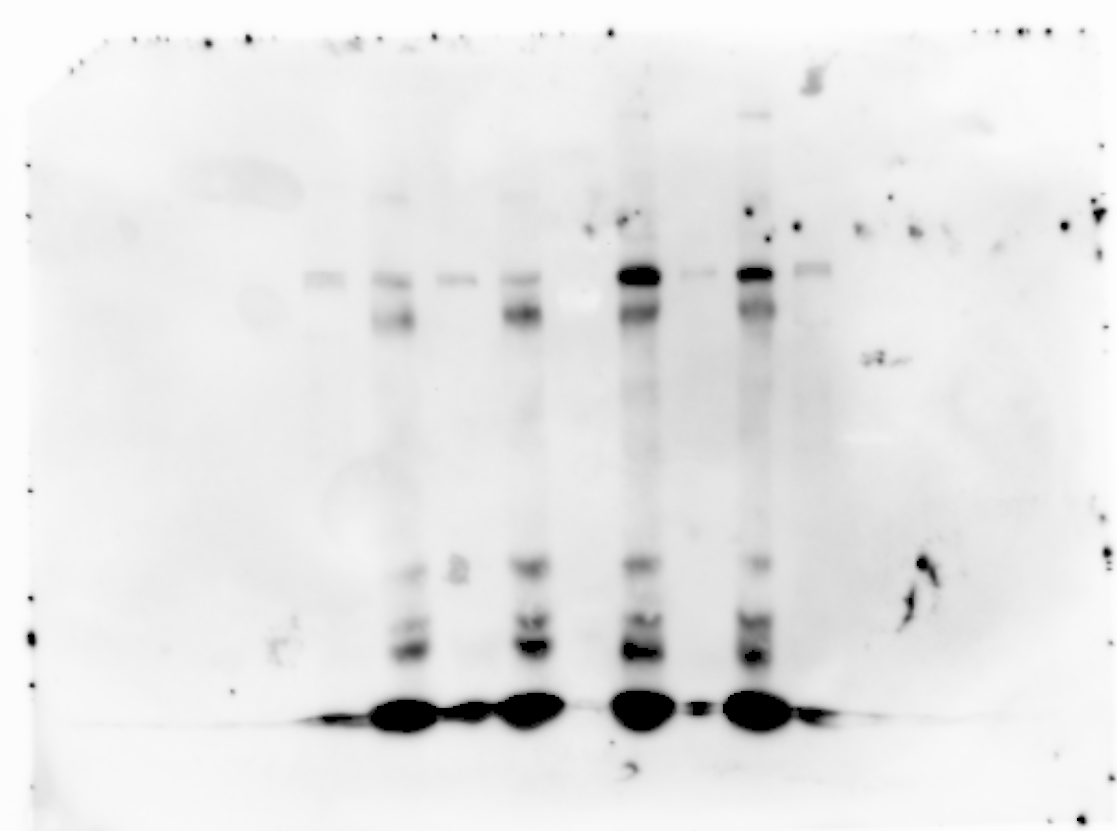


old

old

Old (negative control)

Old (negative control)

young

young

Young (negative contol)

Young (negative contol)


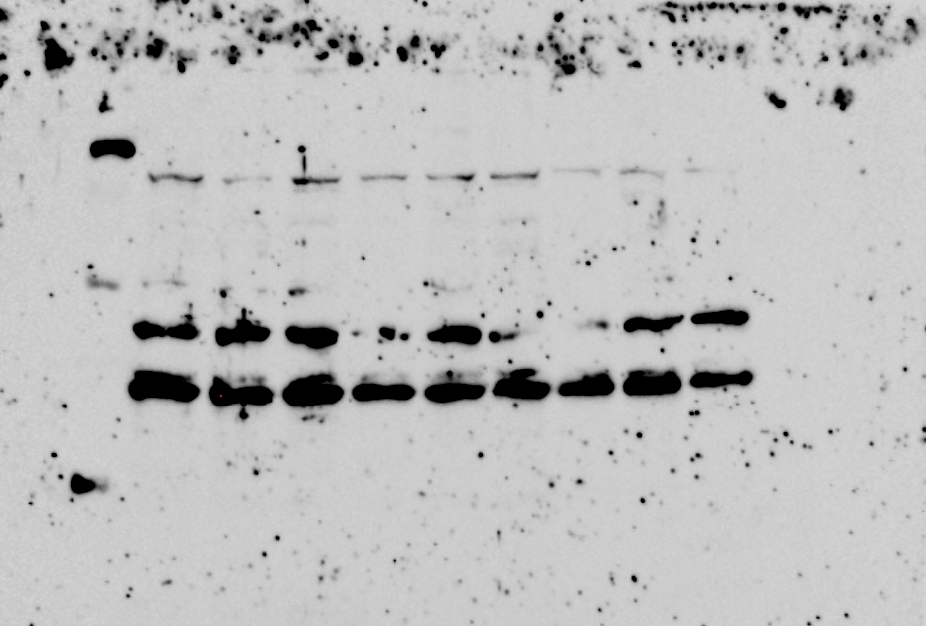


old

young

old

young

old

young

old

young

old

DCG-04 labelling

Cathepsin B


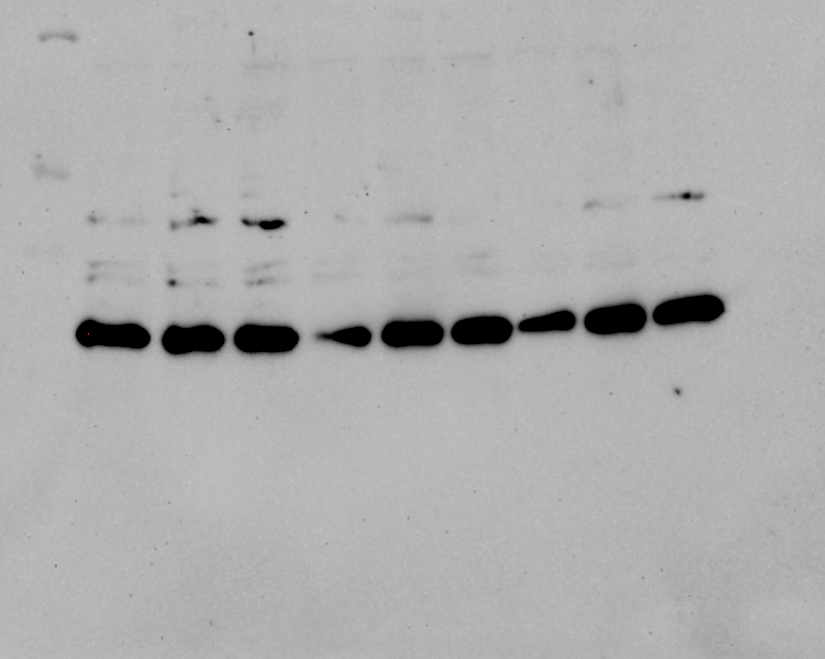


old

young

old

young

old

young

old

young

old

young


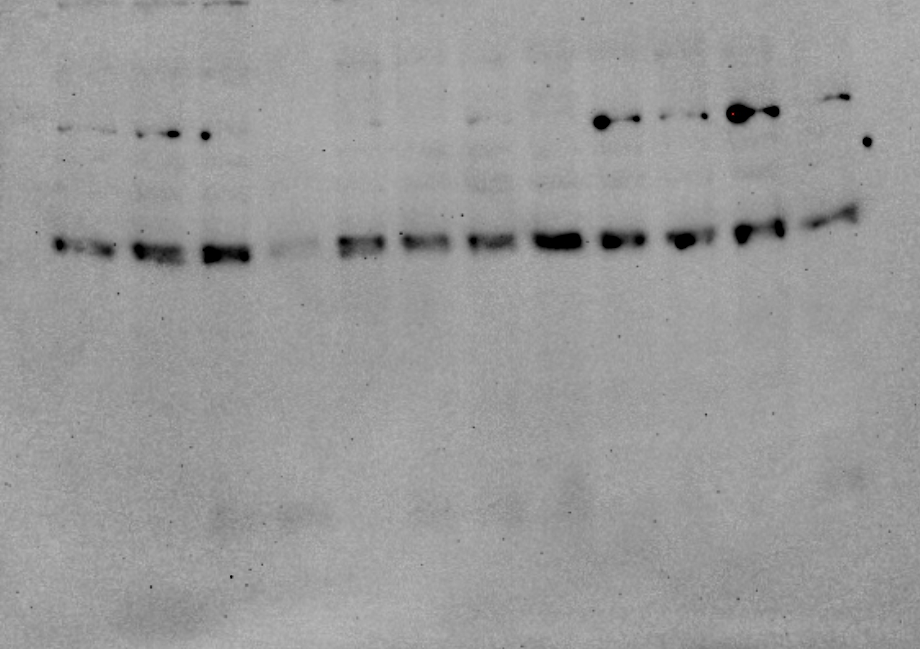


old

old

old

old

young

young

young

young

Cathepsin L

Cathepsin x

**Legend of Fig 1. The activity levels of specific cysteine peptidases in lung tissue extracts, previously marked with DCG-04, from both old and young rats**. (**A**) The proteins extracted from tissue samples of both old and young rats were labeled using the activity-based probe DCG-04. These labeled proteins were then analyzed through protein electrophoresis and subsequent Western blotting, employing streptavidin-horseradish peroxidase detection. (**B**) The analysis encompassed the assessment of calpain-1 content, encompassing both its unprocessed and processed forms, as well as the unprocessed form of cath B/L, processed form of cath B/L, and cath X bands within lung tissue extracts obtained from both young and old rats. A comparison was made between DCG-04 labeled cells and cells labeled only with DCG-04 (Control). (**C**) The avidin pull-down experiment involving cysteine cathepsins in tissue extracts from both old and young rats was conducted following the outlined procedures in the materials and methods section. Proteins conjugated to avidin Sepharose beads were subsequently analyzed through SDS-PAGE and Western blotting using antibodies specifically targeting human cathepsins B, L, and X. (**D**) The comparative analysis of cellular contents ratio for cathepsins B, L, and X in tissue extracts from both old and young rats. The dataset underwent statistical analysis via two-way ANOVA with subsequent Dunnett’s post hoc analysis (*** p < 0.001; n = 5). Statistical computations were performed using GraphPad Prism.

**Fig. 2 Labelling and pulldown of aspartic cathepsins D**


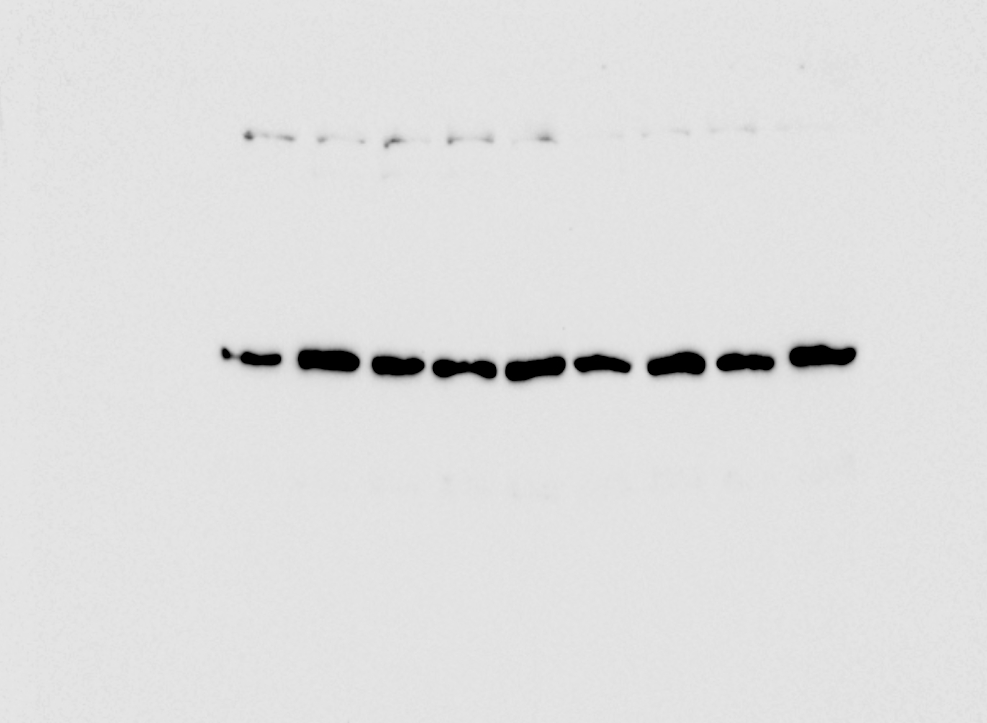

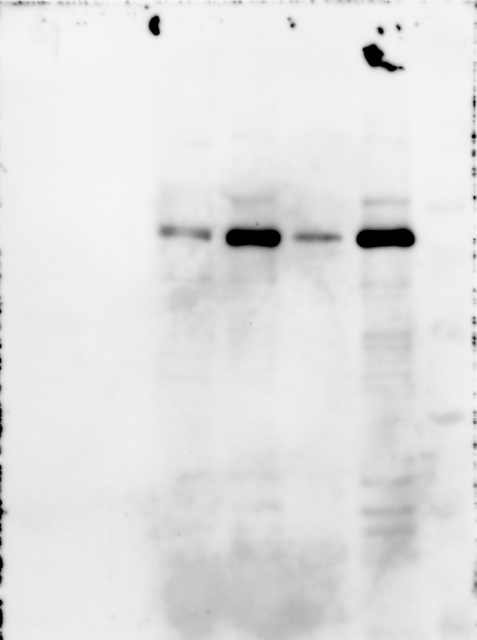


Cathepsin D pulldown

young

old

young

old

young

old

young

old

Pepstatin A-biotin labelling

old

young

**Ligand of Fig 2.** **Displays the activity levels of specific aspartic peptidases in lung tissue extracts previously labeled with pepstatin A-biotin, obtained from both old and young rats**. (**A**) proteins from lung tissue samples of both old and young groups were labeled using pepstatin A-biotin and subjected to protein electrophoresis and subsequent western blotting, detected via streptavidin-horseradish peroxidase. (**B**) Focuses on assessing cath D bands within these lung tissue extracts, comparing the pepstatin-A labeled aspartic peptidases between old and young rats. (**C**) Illustrates the avidin pull-down experiment targeting aspartic cathepsins in tissue extracts from both old and young groups, followed by SDS-PAGE and Western blotting using antibodies specific to human cathepsins D. Lastly, (**D**) Presents the comparative analysis of tissue content ratios for cath D in both old and young rat extracts, analyzed via two-way ANOVA with Dunnett’s post hoc analysis (n = 5) using GraphPad Prism for statistical computations.

**Fig. 3 A. Labelling and pulldown of serine cathepsin G**


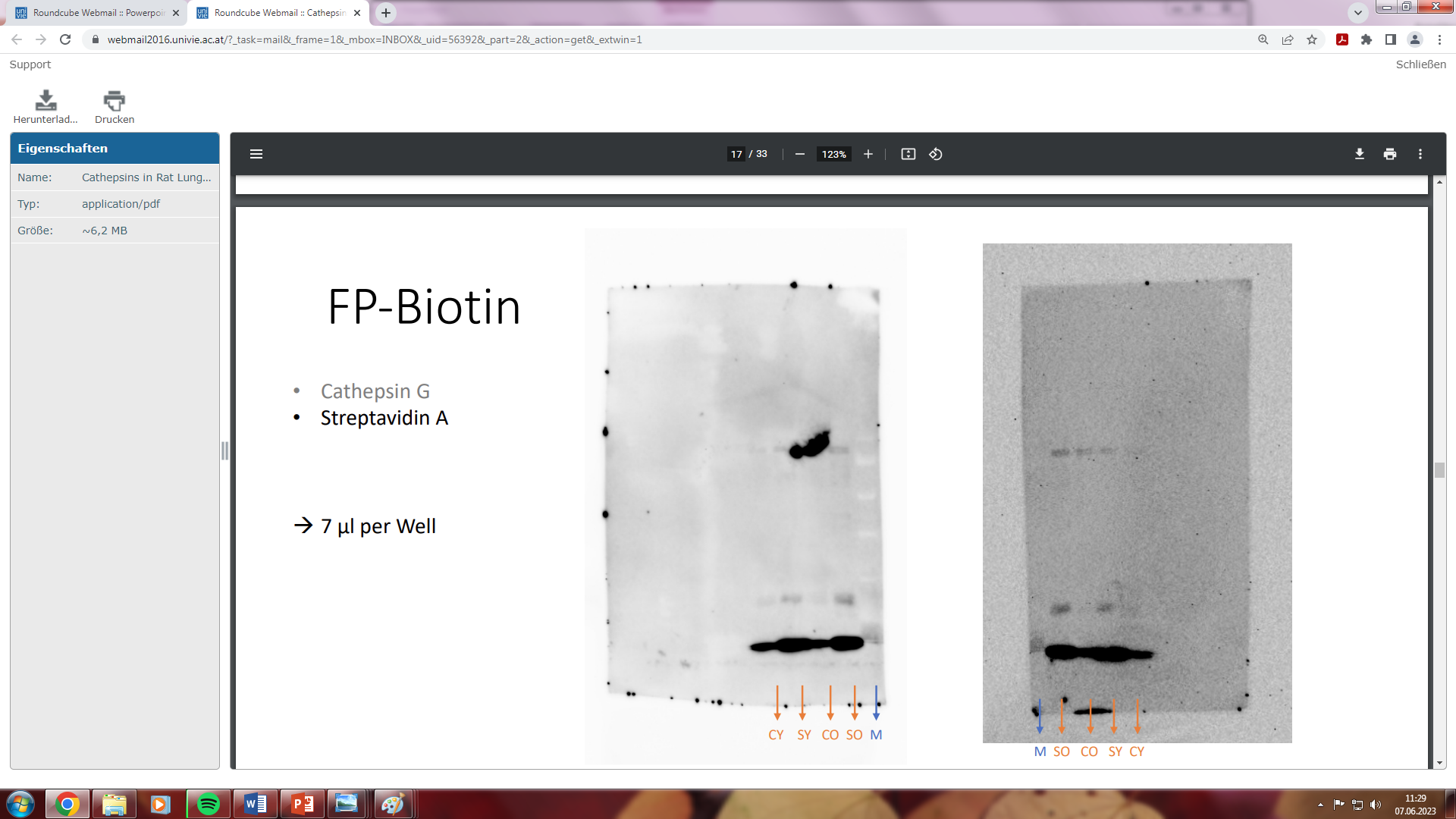


old

young


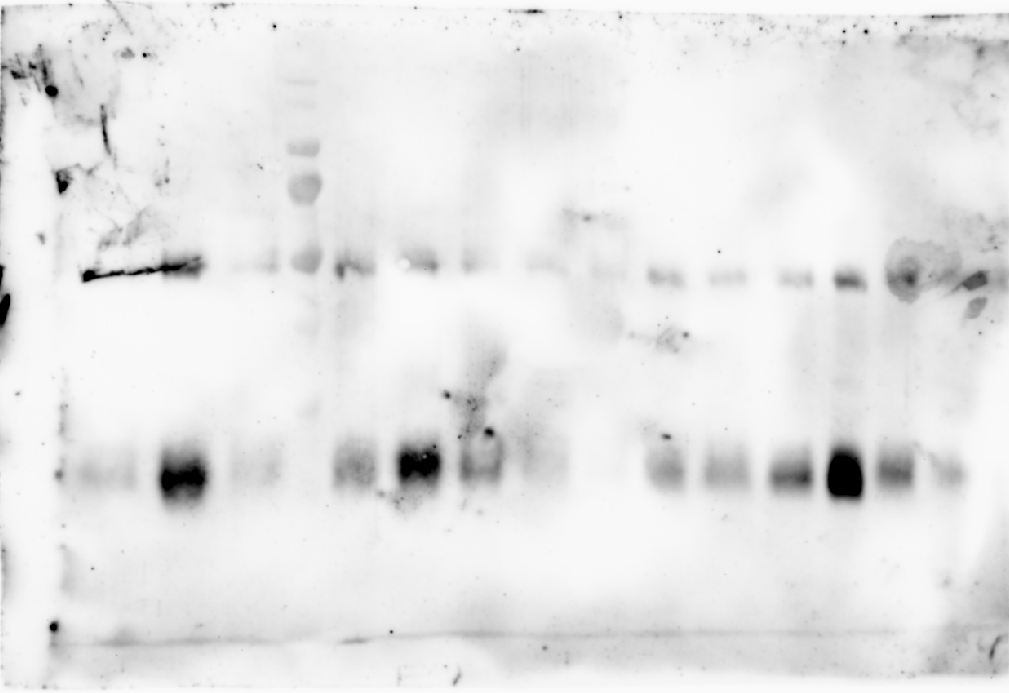


young

old

young

old

old

young

young

young

old

old

young

young

Fig. 3 C

**Ligand of Fig 3.** **Displays the activity levels of specific serine peptidases in lung tissue extracts labeled with FP-biotin from both old and young rats**. (**A**) Proteins from lung tissue samples of both age groups were labeled using FP-biotin, subjected to protein electrophoresis, and analyzed via western blotting using streptavidin-horseradish peroxidase. (**B**) Focuses on comparing the cath G bands in these lung tissue extracts, specifically assessing FP-biotin labeled serine peptidases between old and young rats. (**C**) Illustrates the avidin pull-down experiment targeting serine cathepsins in tissue extracts from both age groups, followed by SDS-PAGE and Western blotting using antibodies specific to human cath G. Lastly, (**D**) Presents a comparative analysis of tissue content ratios for cath G in both old and young rat extracts, analyzed using two-way ANOVA with Dunnett’s post hoc analysis (*** p < 0.001; n = 5) via GraphPad Prism for statistical computations.

**Fig. 4 Zymography experiments showing the metalloproteinase activities in young and old rat tissues**


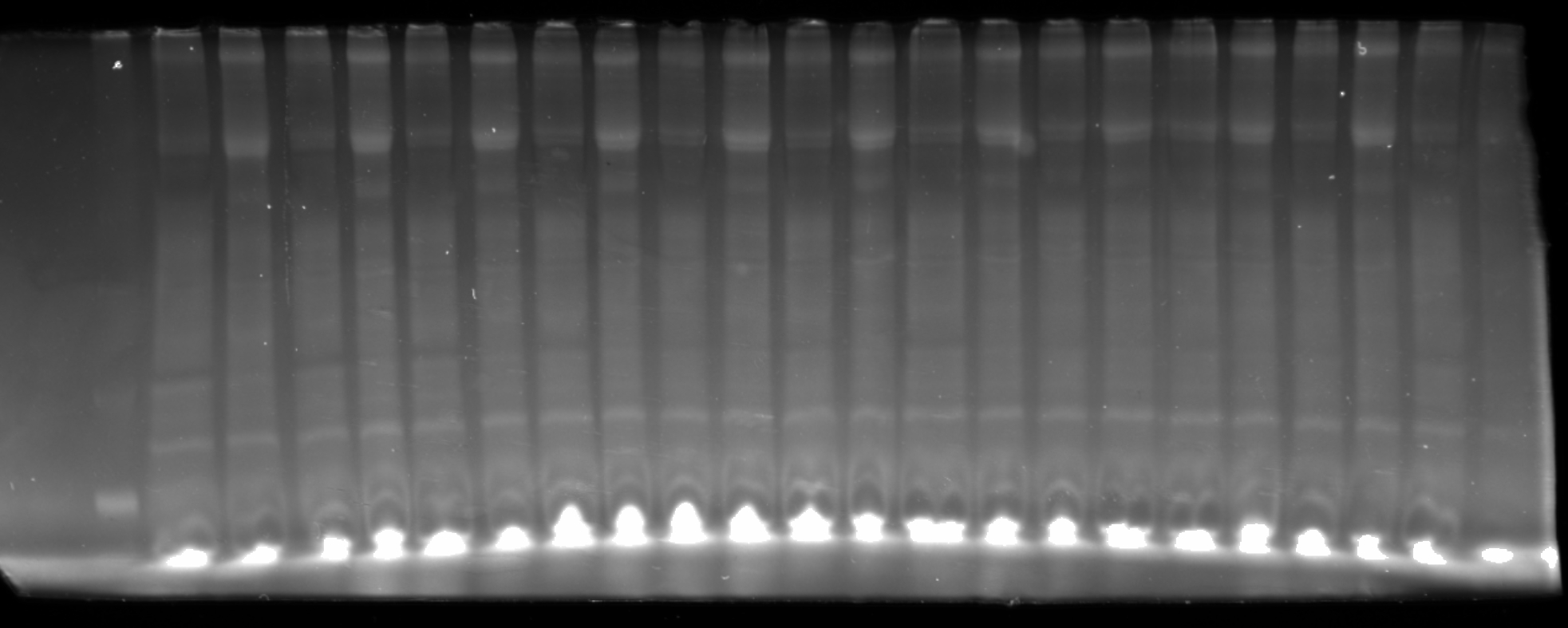


young

young

young

young

young

young

young

young

young

young

old

old

old

old

old

old

old

old

old

old

**Fig. 5 Fibronectin degradation in old and young lungs**

**Ligand of Fig 4**. **MMP-2 and MMP-9 activities in the lung tissues of both old and young rats**. (**A**) Gelatin zymography of MMP-2 and MMP-9 activities in old and young lung tissues. (**B**) Presents a comparative analysis of tissue activity ratios for MMP-2 and MMP-9 in both old and young rat extracts, analyzed using two-way ANOVA with Dunnett’s post hoc analysis (*** p < 0.001; n = 5) via GraphPad Prism for statistical computations.

**Fig.5**
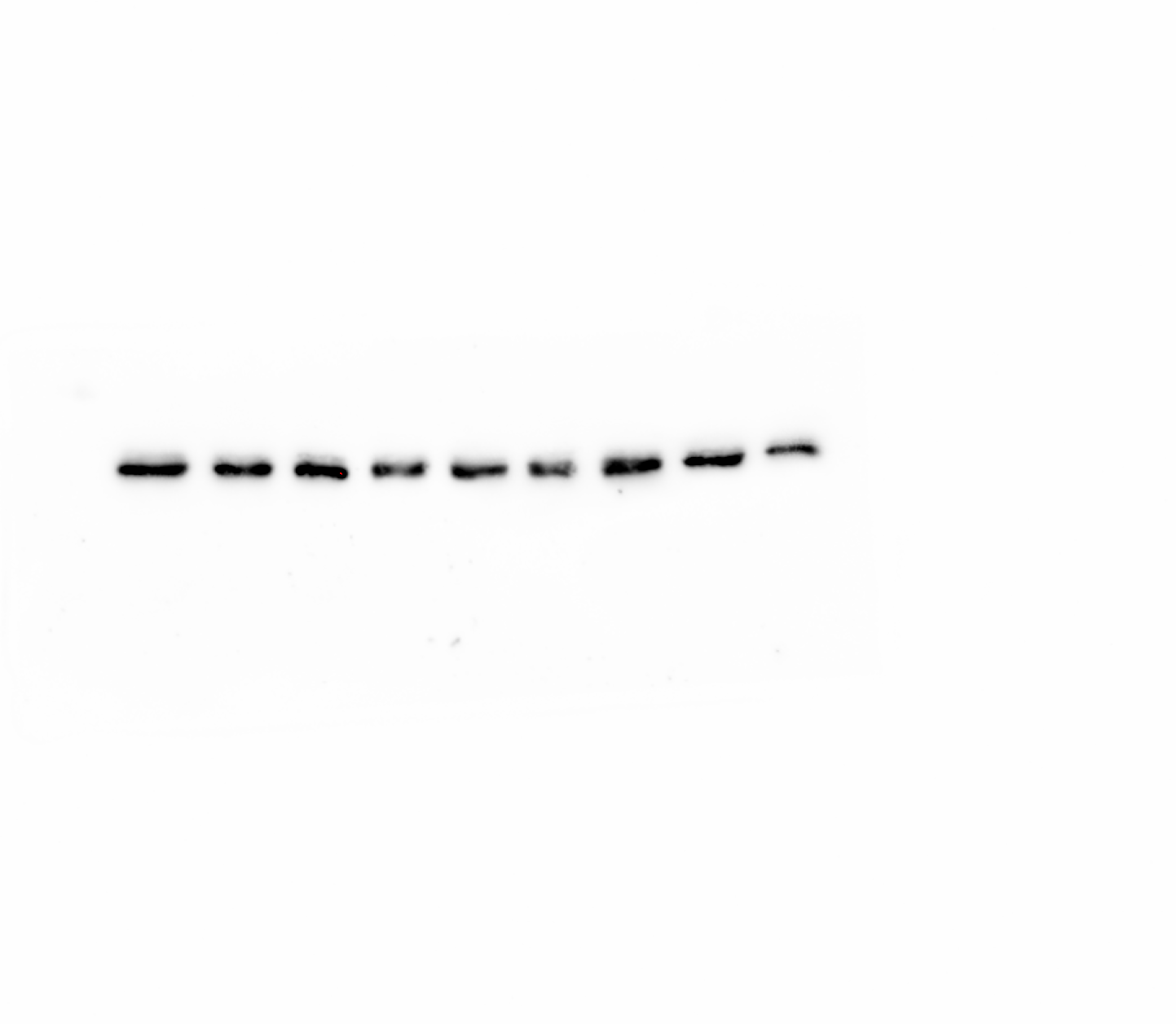


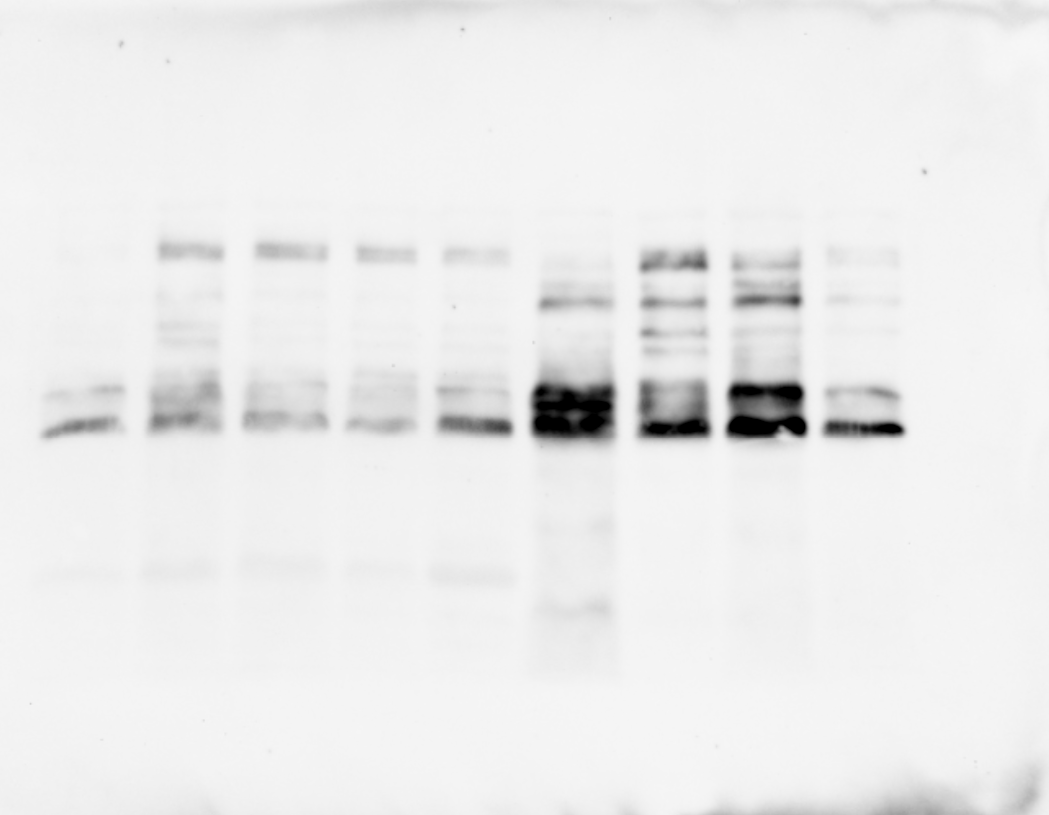


young

young

young

young

old

old

old

old

old

**Ligand of Fig 5.** **Fibronectin degradation analysis**. 20 μg lung tissue proteins of both old and young rats were exposed to SDS-PAGE and Western blotting using antibodies specific to human fibronectin. This experiment was performed five times with similar results.

**Fig.6**


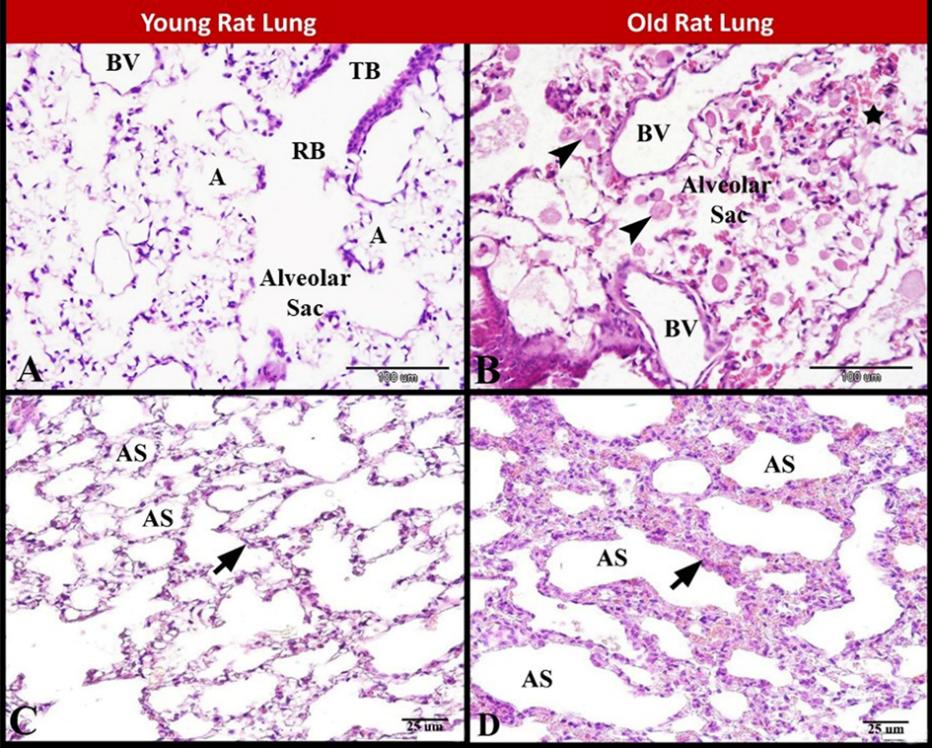


**Ligand of Fig 6.** **Photomicrograph of paraffin sections showing structural changes during ratpulmonary senescence** . **(A**) Young rat lung showing normal terminal bronchiole (TB), respiratory bronchiole (RB), alveolar sac, alveoli (A) and blood vessels (BV). **(B)** Old rat lung showing pulmonary tissue destruction (star), aged blood vessels (BV) and disturbed alveolar sac filled with alveolar macrophages (arrow heads). **(C**) Young rat lung showing normal, small, numerous and evenly distributed air spaces (AS) with normal interstitial tissue (arrow). (**D)** Old rat lung showing few large irregular air spaces (AS) with thickening and abundant interstitial tissue (arrow). Stain: H and E, Scale bar in A & B = 100 µm, Scale bar in C & D = 25 µm.

**Fig.7**

**
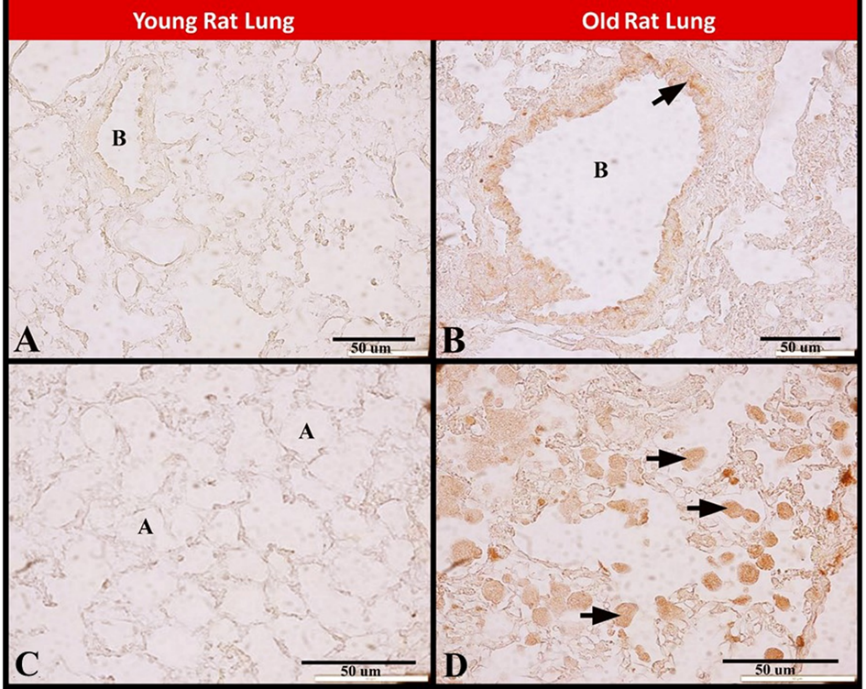
**

**Ligand of Fig 7.** **Photomicrograph of paraffin sections showing immunoexpression of cath G during rat pulmonary senescence** . **(A, C)** Young rat lung showing no or slight cath G immunostaining in pulmonary bronchioles in **A** and pulmonary alveoli in **C**. **(B, D)** Old rat lung showing abundant strong cath G immunostaining in pulmonary bronchioles (arrow) in **B** and in alveolar macrophages (arrow) in **D**. Pulmonary bronchioles (B) and pulmonary alveoli (A). Scale bar in A-D = 50 µm.

**Fig.8**


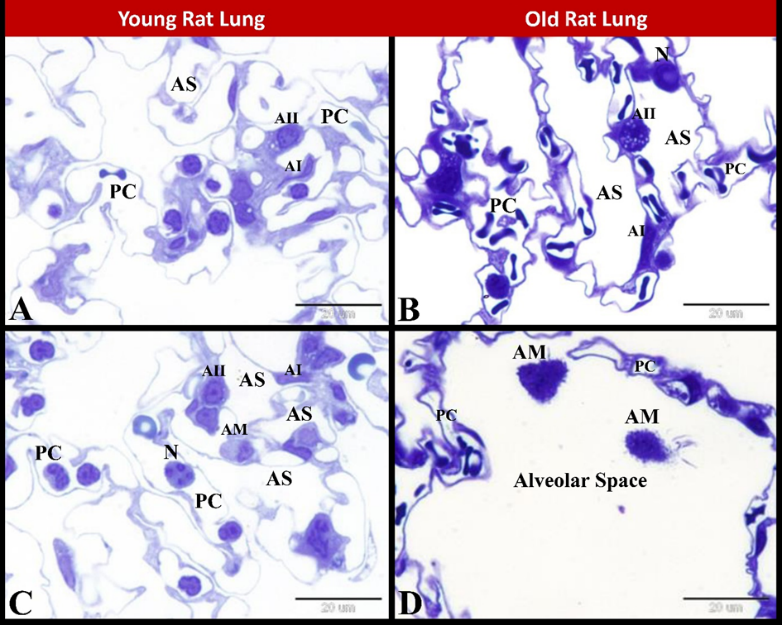


**Ligand of Fig 8.** Photomicrograph of semi-thin sections showing structural changes during rat pulmonary senescence. **(A, C)** Young rat lung showing normal small alveolar space, alveolar type 1 and alveolar type 2 cells and normal slightly wide pulmonary capillaries with leucocytes as neutrophils. Note the alveolar macrophage in **C**. **(B, D)** Old rat lung showing in **B** widening of alveolar space, aged alveolar type 1 and alveolar type 2 cells, aged narrow pulmonary capillaries and extra capillary neutrophil in the alveolar space. in **D** showing large wide alveolar space contained aged alveolar macrophage and surrounded by aged narrow pulmonary capillaries. Alveolar space (AS), alveolar type 1 (AI), alveolar type 2 (AII), pulmonary capillaries (PC), neutrophils (N) and alveolar macrophage (AM). Stain: Toluidine blue, Scale bar in A-D = 20 µm.

**Fig.9**

**
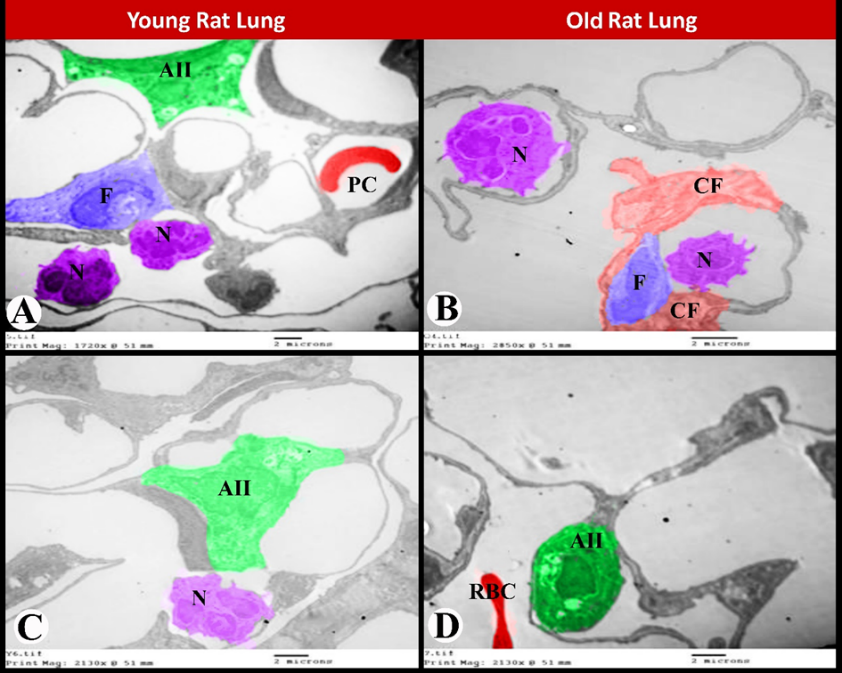
**

**Ligand of Fig 9. Transmission electron micrograph of ultrathin sections showing ultrastructure changes during rat pulmonary senescence (A, C)** Young rat lung showing normal alveolar type 2 cells and normal slightly wide pulmonary capillaries, interstitial fibroblasts and normal neutrophils with its characteristic segmented nucleus and granules. **(B, D)** Old rat lung showing aged neutrophils with its characteristic large size and numerous pseudopodia, interstitial fibroblasts surrounded by abundant collagen fibers, aged alveolar type 2 cells and extravasated red blood cells inside the alveolar space. Alveolar type 2 (AII), pulmonary capillaries (PC), neutrophils (N), fibroblasts (F), collagen fibers (CF) and red blood cells (RBC). Scale bar in A-D = 2 µm.

**Row Data for the Figures**

**Table 1 a for Figure 1 C and D**

| Fig 1 C |  |  |  |  |  | Fig. 1 D |  |
| --- | --- | --- | --- | --- | --- | --- | --- |
|  |  |  |  |  |  |  |  |
|  | old | young |  |  |  | old | young |
| calpain 1 unprocessed | 45 | 50 |  |  | cathepsin L | 88 | 112 |
|  | 54 | 49 |  |  |  | 98 | 86 |
|  | 40 | 42 |  |  |  | 104 | 88 |
|  | 59 | 57 |  |  |  | 86 | 95 |
|  |  |  |  |  |  |  |  |
|  |  |  |  |  |  |  |  |
| Calpain 1 processed | 22 | 20 |  |  | cathepsin B | 161 | 180 |
|  | 25 | 27 |  |  |  | 172 | 134 |
|  | 21 | 22 |  |  |  | 155 | 166 |
|  | 29 | 25 |  |  |  | 151 | 150 |
|  |  |  |  |  |  |  |  |
| procathepsin B/L | 15 | 20 |  |  |  |  |  |
|  | 17 | 14 |  |  | cathepsin X | 60 | 50 |
|  | 18 | 16 |  |  |  | 66 | 52 |
|  | 17 | 19 |  |  |  | 50 | 55 |
|  |  |  |  |  |  | 52 | 49 |
| cathepsin B/l | 11 | 10 |  |  |  |  |  |
|  | 14 | 15 |  |  |  |  |  |
|  | 10 | 12 |  |  |  |  |  |
|  | 12 | 10 |  |  |  |  |  |
|  |  |  |  |  |  |  |  |
| cathepsin X | 34 | 29 |  |  |  |  |  |
|  | 39 | 40 |  |  |  |  |  |
|  | 30 | 37 |  |  |  |  |  |
|  | 35 | 36 |  |  |  |  |  |

Table 2 a for Figure 2 C and D

| Fig.2C |  |  | Fig. 2D |  |
| --- | --- | --- | --- | --- |
|  |  |  |  |  |
| old | young |  | old | Young |
| 29 | 26 |  | 68 | 70 |
| 24 | 27 |  | 84 | 81 |
| 28 | 31 |  | 76 | 60 |
| 31 | 29 |  | 67 | 62 |

Table 3 a for Figure 3 C and D

| Fig. 3A |  |  | Fig. 3B |  |
| --- | --- | --- | --- | --- |
|  |  |  |  |  |
| old | young |  | old | young |
| 55 | 24 |  | 90 | 37 |
| 60 | 19 |  | 99 | 42 |
| 48 | 22 |  | 88 | 36 |
| 56 | 27 |  | 83 | 39 |

Table 4 a for Figure 4 b

| Fig. 4 b |  |  |  |  |
| --- | --- | --- | --- | --- |
| MMP-9 |  |  | MMP-2 |  |
| old | young |  | old | young |
| 42 | 26 |  | 48 | 29 |
| 45 | 29 |  | 50 | 35 |
| 49 | 34 |  | 52 | 39 |
| 52 | 31 |  | 55 | 27 |
